# The preceding root system drives the composition and function of the rhizosphere microbiome

**DOI:** 10.1186/s13059-020-01999-0

**Published:** 2020-04-06

**Authors:** Yi Zhou, David R. Coventry, Vadakattu V. S. R. Gupta, David Fuentes, Andrew Merchant, Brent N. Kaiser, Jishun Li, Yanli Wei, Huan Liu, Yayu Wang, Shuheng Gan, Matthew D. Denton

**Affiliations:** 1 School of Agriculture, Food and Wine, The University of Adelaide, Glen Osmond, SA 5064 Australia; 2 China-Australia Joint Laboratory for Soil Ecological Health and Remediation, The University of Adelaide, Glen Osmond, SA 5064 Australia; 3 CSIRO Agriculture and Food, Glen Osmond, SA 5064 Australia; 4 School of Life and Environmental Sciences, University of Sydney, Brownlow Hill, NSW 2570 Australia; 5 Shandong Provincial Key Laboratory of Applied Microbiology, Ecology Institute, Qilu University of Technology (Shandong Academy of Sciences), Shandong, 250013 China; 6 BGI-Shenzhen, Shenzhen, 518083 Guangdong China

**Keywords:** Soil microbiome, Metagenome, Tillage, Agricultural system, Root

## Abstract

**Background:**

The soil environment is responsible for sustaining most terrestrial plant life, yet we know surprisingly little about the important functions carried out by diverse microbial communities in soil. Soil microbes that inhabit the channels of decaying root systems, the detritusphere, are likely to be essential for plant growth and health, as these channels are the preferred locations of new root growth. Understanding the microbial metagenome of the detritusphere, and how it responds to agricultural management such as crop rotations and soil tillage, is vital for improving global food production.

**Results:**

This study establishes an in-depth soil microbial gene catalogue based on the living-decaying rhizosphere niches in a cropping soil. The detritusphere microbiome regulates the composition and function of the rhizosphere microbiome to a greater extent than plant type: rhizosphere microbiomes of wheat and chickpea were homogenous (65–87% similarity) in the presence of decaying root (DR) systems but were heterogeneous (3–24% similarity) where DR was disrupted by tillage. When the microbiomes of the rhizosphere and the detritusphere interact in the presence of DR, there is significant degradation of plant root exudates by the rhizosphere microbiome, and genes associated with membrane transporters, carbohydrate and amino acid metabolism are enriched.

**Conclusions:**

The study describes the diversity and functional capacity of a high-quality soil microbial metagenome. The results demonstrate the contribution of the detritusphere microbiome in determining the metagenome of developing root systems. Modifications in root microbial function through soil management can ultimately govern plant health, productivity and food security.

## Background

The establishment of a gene catalogue aids in the understanding and identification of options for potentially manipulating microbial communities in complex environments. Global gene catalogues of microbiomes have been established from the human gut [1], from mouse [2] and pig [3] gut, from the human skin [4] and from ocean water [5]. Although efforts have been made to capture a microbial gene catalogue from global topsoils [6], prairie and cornfield soils [7] and citrus roots [8], observations indicate that insufficient coverage and under-sampling can affect the estimation of the enormous functional (gene) potential of soil microbiomes. With such complexity, ultradeep sequencing (e.g. 0.6 to 1 Tb required for complete soil genome coverage [9]) and bioinformatic assemblages are required to draw a saturated gene catalogue of a defined soil metagenome [7].

Spatial heterogeneity in soil microbiomes is driven by microsites, the biologically relevant spheres of influence such as rhizosphere, detritusphere and porosphere, each with distinct physico-chemical properties operating at different spatial scales [10]. Microbiomes in the rhizosphere, a zone close (typically < 2 mm) to the plant root, are strongly influenced by plant roots, and the rhizosphere generally has much higher chemical and biological activity than bulk soil, due to exudates from roots [11–14]. Plants selectively influence the composition and activity of the rhizosphere microbiome through chemical communication and provision of carbon and nutrients. Rhizosphere microbiota play important roles in improving the growth of host plants, through the regulation of plant essential functions including nutrient cycling and uptake, root and shoot growth, disease suppression and induced systemic resistance and abiotic stress tolerance [15–18]. The composition and function of the rhizosphere microbiome have been evaluated using reconstructed soils in controlled conditions [19–21] and in on-farm environments [22, 23]. Crop management practices such as tillage, residue retention and crop rotation are crucial components in the functioning of an agroecosystem, but are often neglected when studying the dynamics of rhizosphere microbiota, in particular during the establishment of rhizosphere microbial communities in early seedling growth phases in no-till or disrupted soil profiles. In view of the significant influence that the detritusphere (soil surrounding the decaying root from the previous crop) can have on soil microbial communities, it is hypothesised that early seedling rhizosphere communities would be influenced by the detritusphere microbiomes.

Microbiomes in agricultural systems are altered by both crop and soil management. Growing annual crops invariably involves some level of soil disturbance from tillage and sowing operations, along with variation in the sequence of crops grown (crop rotations) [24]. The practice of no tillage (NT) is used globally in agriculture and limits the destruction of soil structure and retains much of the prior rotation crop’s root residue and microsite structure (aggregates, pores, detritusphere etc.), compared with historical conventional tillage (CT) practices that disrupt the soil structure [25, 26]. The use of NT has provided the opportunity for the development of more intensive crop rotation practices, especially in soils with structural problems. Importantly, the NT system is characterised by an abundance of historic root channels that contain the residues of antecedent root systems referred to here as the detritusphere. The decaying root material typically contains more active microbes than the bulk soil [27]. In the NT systems, root channels are typically maintained. As a consequence, a large portion of the roots of a newly established crop occupy previously established root channels due in part to lower mechanical resistance [28, 29], commonly categorised as part of a “sense-by-growth” mechanism [30]. Therefore, in an NT-intensive cropping system, the formation of the rhizosphere microbiome associated with the new crop’s active roots may be in part determined by the microorganisms existing in the detritusphere, but to date, little is known about the detailed microbiome structure associated with these microsites.

The early seedling phase is crucial for plants in establishing the rhizosphere and endosphere microbiomes, as it is the start of the plant root selecting specific members of the soil microbiome [13]. Microbiome composition of seedlings has been shown to influence the early growth of wheat seedlings under no-till cropping systems [31]. In legumes, specific root exudates signal the bacterial population to encourage successful symbiotic events with rhizobia [32]. These examples demonstrate the presence of a dynamic interaction between soil habitat conditions, plant root and soil microbiome with potential consequences to plant growth, health and overall crop performance.

Here, our study uses the seedlings of wheat (*Triticum aestivum* L.) and chickpea (*Cicer arietinum* L.), representing cereal and legume crops, respectively, to evaluate the interaction of plants with differential root exudation and cropping management (+ and − decaying root) on shaping the rhizosphere microbiome composition. Chickpea and wheat have contrasting root exudation, as chickpea is characterised by a great quantity of organic and amino acid root exudates compared with wheat [33]. In the present study, we compare the function and taxonomic structure of the rhizosphere microbiome between these different crops in the presence (+) and absence (−) of decaying root. In particular, we highlight the role of the detritusphere and its decaying root in influencing the development of the rhizosphere microbiomes of a cereal and a legume seedling.

## Results

### The physical, chemical and biological properties of niches

Here, we collected the intact soil cores with decaying root (+DR), and the second sample of repacked soils with removed decaying root (−DR) from the same field. In each of these +DR and −DR treatments, wheat or chickpea plants were grown in a controlled environment (Additional file 1: Figure S1). In addition, unplanted cores with + and −DR were used as the control for detritusphere and bulk soil sampling, respectively (Additional file 1: Table S1). Therefore, most of the soil physical and chemical properties were similar between + and −DR (Additional file 1: Table S2). The existence of the DR and its physiochemical properties was the key factor evaluated for its effect in influencing the rhizosphere microbiome structure and function of the living roots compared with the non-DR control.

**Fig. 1:**
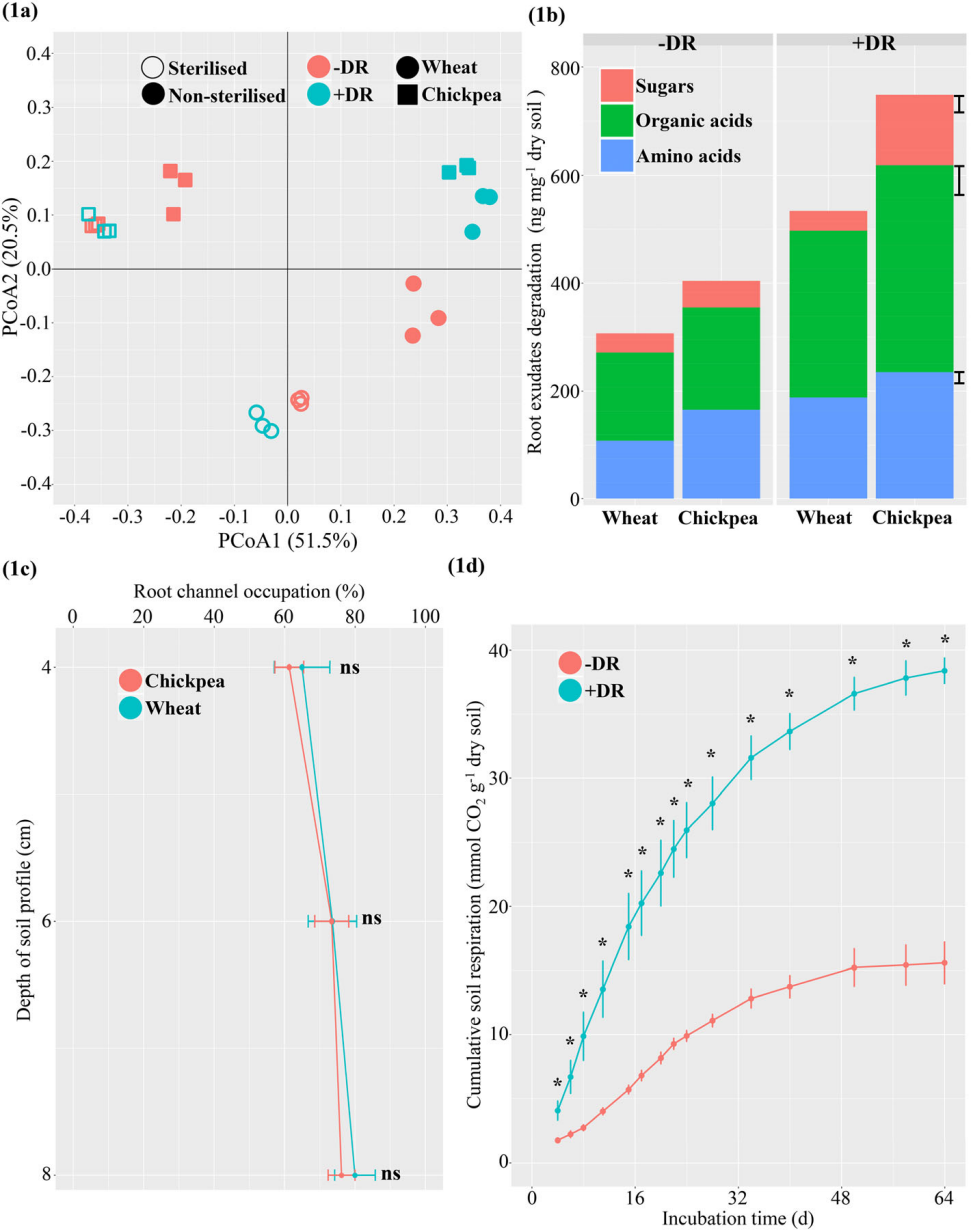
The root-soil characterisation of wheat and chickpea influenced by decaying root (DR). **a** Root exudation compounds (RECs) were collected from wheat and chickpea rhizosphere growing in the sterilised and non-sterilised soil under + and −DR. Principal coordinate analysis (PCoA) was based on Bray-Curtis distance between the relative abundance of 26 detected RECs. The percentage of variance explained by principal components 1 and 2 is shown in parenthesis. **b** REC degradation by rhizosphere microbiome. The 26 detected RECs were clustered into three chemical groups. The error bar is the least significant difference (LSD) at *P* = 0.05. **c** Decaying root channel occupation by the living root of wheat and chickpea. **d** Cumulative soil respiration by incubating the unplanted pots with + and −DR. ANOVA test at *P* < 0.05 showed that treatment effects on REC degradation and soil reparation at every measured time point were significant (*), but not significant for the root channel occupation (ns). Bars indicate the standard error at *P* = 0.05

Differentiation of root exudate composition (based on the 26 detected root exudates) between the studied niches had a significant three-way interaction of plant type, decaying root (+ and −) and sterilisation (+ and −), based on *P* < 0.01 on permutational multivariate ANOVA (PERMANOVA) test using Bray-Curtis distance. Under sterilised conditions, the separation was reliant on plant type whereas under non-sterilised conditions, the plant type effect was stronger for −DR but not for +DR (Fig. 1a). This suggests that the existence of decaying root masked the plant type effect on root exudate metabolism.

Rhizosphere root exudate degradation was calculated by a reduction in root exudate concentrations from sterilised to non-sterilised conditions (Fig. 1b). The degradation of root exudates by the rhizosphere microbiome in +DR was greater than for −DR (*P* < 0.01). The chickpea rhizosphere metabolised more root exudates than wheat under both + and −DR. Additionally, in the living soil, chickpea without DR formed an acid rhizosphere compared with bulk soil and wheat rhizosphere (*P* < 0.05, Table 1), but in the presence of DR, there were no significant differences in rhizosphere pH between chickpea and other rhizo-detritusphere niches.

**Table 1:** Chemical and biological properties of plant-soil niches. Measured traits include rhizosphere soil pH, microbial number and the diversity of microbial functions and taxa. OTU and gene were used as a basic unit to estimate diversity. Soil samples for the measurements were collected from the wheat and chickpea rhizosphere under + and − decaying root (+ and −DR). Bulk soil of −DR and rhizosphere of the decaying root were also sampled. The same letter within one row indicated no significant difference based on the least significant difference (LSD) at *P* < 0.05

	+DR			−DR			
	Detritusphere (decaying root)	Wheat rhizosphere	Chickpea rhizosphere	Bulk soil	Wheat rhizosphere	Chickpea rhizosphere	LSD
Rhizosphere soil pH							
Sterilised	6.6a	6.7a	4.5b	6.8a	6.6a	4.4b	0.7
Non-sterilised	6.7a	6.8a	5.9ab	6.6a	6.7a	5.3b	0.8
Microbial number							
Bacteria (copy number × 10^6^ g^−1^ soil)	6032a	6332a	3760b	2490c	2900c	3036c	548
Fungi (copy number × 10^6^ g^−1^ soil)	50b	60a	22c	20c	26c	22c	10
Bacteria:Fungi	124a	107a	195a	136a	122a	149a	ns
Beta-diversity (Bray-Curtis distance between wheat and chickpea rhizosphere)							
OTUs		0.2384b			0.3681a		0.0222
Genes		0.2689b			0.4107a		0.0025
Alpha-diversity (Shannon index)							
OTUs	5.870a	5.868a	5.820a	5.319c	5.382bc	5.410b	0.069
Genes	14.521b	14.564a	14.501b	14.464c	14.465c	14.379d	0.018

Regarding the influence of rhizosphere from the detritusphere, there was a close contact between the fresh root growth and residue root in the +DR treatment. For both wheat and chickpea, approximately 60–80% of the new roots followed the channels of the previous root residue (Fig. 1c). In +DR pots where fresh roots closely interacted with residue root channels, there was a greater root distortion rate compared to that in the −DR pots (*P* < 0.01, Additional file 1: Figure S2, visualised in Figure S1).

The bacteria number and activity (cumulative CO_2_–C evolved) were higher (double; *P* < 0.01) in the rhizosphere under +DR condition than in −DR (Table 1 and Fig. 1d). Under +DR, only wheat rhizosphere and the detritusphere treatments harboured more fungi than −DR, while compared with bacteria, fungi was a minor proportion of the microbial community, contributing only 1/100–1/200 the copy number of bacteria (Table 1).

Hence, the existence of decaying root changed the rhizosphere metabolism, root growth and rhizobiome activities of the living roots, and played a significant role in shaping the plant rhizosphere microbiomes.

### Microbial taxonomic and functional composition between niches

**Fig. 2:**
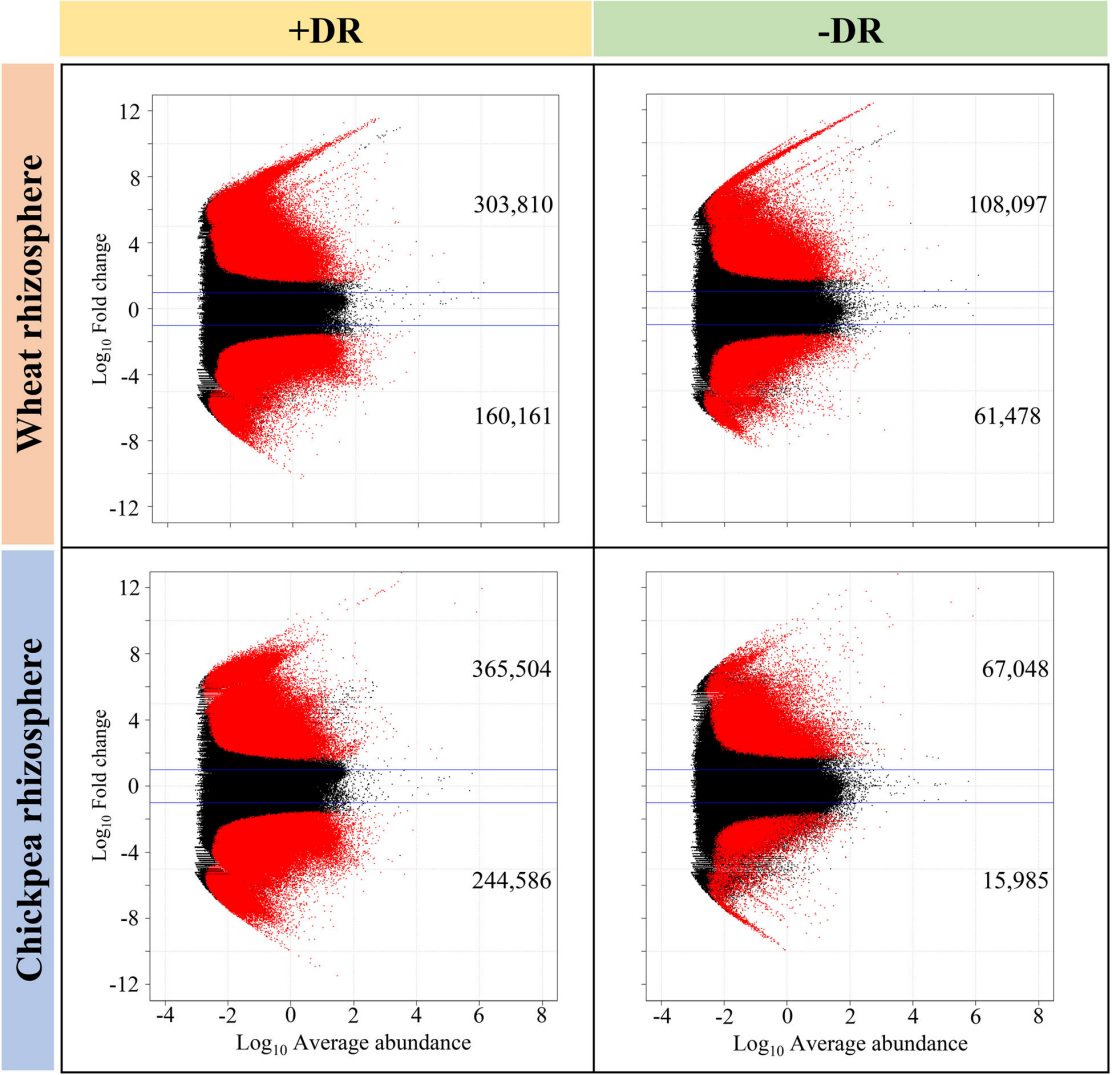
Differentially abundant genes from the rhizosphere microbiome of wheat and chickpea under + and − decaying root (DR). Enrichment and depletion of genes (red spots) to bulk soil were defined according to log2-fold-change > 1 and the FDR adjusted *P* value < 0.01

Based on the amplicon sequencing of 16S rRNA genes and metagenome sequencing data, differential abundance analysis was conducted using unplanted bulk soil without decaying root material as the control. We compared the differences in the abundance of individual assembled operational taxonomic units (OTUs), non-redundant genes and KOs between rhizosphere niches (including + and −DR) and bulk soil. The enriched and depleted OTUs, non-redundant genes and KOs for each rhizosphere of different crop types under + and −DR were identified based on log2-fold-change > 1 and the FDR adjusted *P* value < 0.01 using 3 replicates (Fig. 2).

**Fig. 3:**
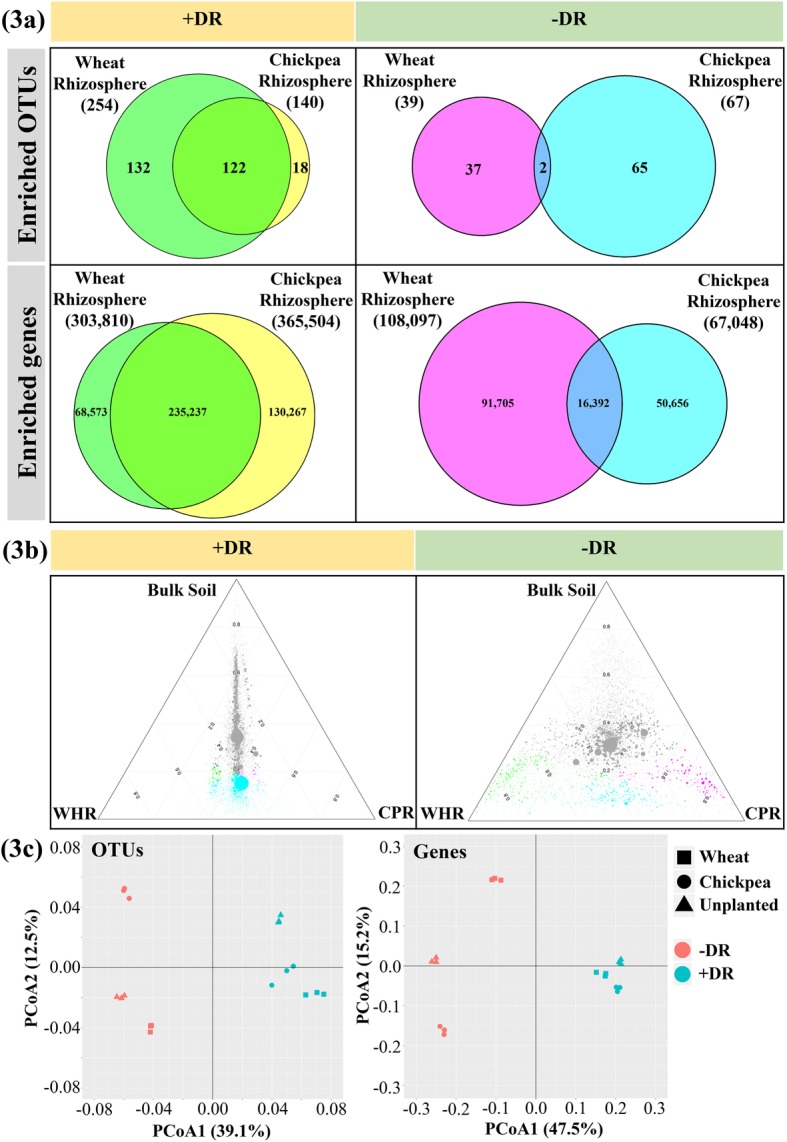
Comparing the taxonomic and functional structure of rhizosphere microbiome between wheat and chickpea growing under + and − decaying root (DR). **a** The number of enriched OTUs and genes shared between wheat and chickpea under + and − DR. **b** Ternary plot included all the detected KOs between bulk soil and the rhizosphere of wheat (WHR) and chickpea (CPR) under + and − DR. Each circle indicates one KO. The size of each circle indicates its relative abundance weighted by the average. Each circle’s position is determined by the contribution of bulk soil and two plants’ rhizospheres. Green circles indicate the enriched KOs by wheat rhizosphere compared with bulk soil (log2-fold-change > 1 and the FDR adjusted *P* value < 0.01). Magenta circles indicate the enriched KOs by chickpea rhizosphere. Cyan circles indicate the enriched KOs by both wheat and chickpea rhizosphere. **c** Principal coordinate analysis (PCoA) was based on Bray-Curtis distance between the samples using the normalised abundance of OTUs and genes. The percentage of variance explained by principal components 1 and 2 is shown in parenthesis. The unplanted control in −DR referred to bulk soil, and the soil surrounding the decaying root from unplanted control in +DR was the detritusphere soil

The taxonomy and functional gene composition of the rhizosphere microbiome between wheat and chickpea were compared under + and −DR (Fig. 3). The results demonstrate a high degree of taxonomic and functional similarity of rhizosphere microbiota between these two crops when grown under +DR, while alternatively under –DR, significant variation between wheat and chickpea for both the rhizosphere microbiome composition and function was observed. This conclusion is supported by the results from three different types of statistical analysis (Fig. 3). Firstly, when decaying root existed, wheat- and chickpea-enriched OTUs and genes shared a large proportion of overlap, which represented around 48–77% and 65–87% of the wheat and chickpea individually enriched, respectively (Fig. 3a). In the absence of decaying root, the number of overlapped OTUs and genes enriched by both crops became fewer, with about 5–14% and 3–24% for wheat and chickpea, respectively (Fig. 3a). The results of depleted OTUs and genes were similarly influenced by plant type and decaying root interactions (Additional file 1: Figure S3a).

Under the +DR condition, the contribution to the abundance of each KO and OTU was almost equal between wheat and chickpea, as indicated by the distribution of most of the KOs (Fig. 3b) and OTUs (Additional file 1: Figure S3b) which were distant from both wheat and chickpea corners in the ternary plot. Given that it was difficult to present the large number of genes clearly in the ternary plot, genes were merged into KOs based on their functional similarity in the KEGG database, and KOs were considered as the basic unit to generate the functional ternary plot. In +DR, there were only 9 and 16 out of 5289 KOs, with over 60% counts contributed by the wheat or chickpea rhizospheres, respectively (Fig. 3b). In contrast, in the −DR system, plant type showed a considerable influence on the abundance of KOs: the numbers of KOs with over 60% counts contributed by plant rhizosphere were 621 and 256 out of 5289 for wheat and chickpea rhizospheres, respectively (Fig. 3b).

Principal coordinate analyses (PCoA) on OTUs and genes showed that plant treatments under +DR were closely grouped, but were clearly separated under −DR, especially for gene composition (Fig. 3c). When decaying root and plant type were constrained as two factors, PERMANOVA based on Bray-Curtis distance revealed that decaying root accounted for the majority of variation (about 41% for OTUs and 48% for genes composition, *P* < 0.01, permutations = 999, Additional file 1: Table S3). The effects of individual factors and their interaction were significant (*P* < 0.01) on gene composition, while decaying root × plant type interaction did not show a significant effect on the microbial taxonomic structure. Bray-Curtis distance between wheat and chickpea rhizobiome was shorter under +DR than −DR (*P* < 0.01, Table 1).

Aside from comparing the rhizosphere microbiome between two crops under + or −DR, we studied the enriched and depleted microbial genes (relative to bulk soil) in the detritusphere (soil surrounding the decaying root with no live crops planted). The rhizospheres of wheat and chickpea growing in the presence of decaying roots shared a large proportion of differential genes (both enriched and depleted) with those observed in the detritusphere sample. Of the enriched and depleted genes in the detritusphere sample, 44–53% were observed in wheat or chickpea rhizospheres in +DR, indicating a significant overlap (Additional file 1: Figure S4). Furthermore, under –DR, the wheat rhizosphere microbiome shared a larger number of enriched genes and OTUs with detritusphere than in the chickpea rhizosphere vs detritusphere (Additional file 1: Figure S4).

In addition to beta-diversity variation between sampled niches, the alpha-diversity of microbiomes such as the Shannon index of OTUs and genes was higher in the rhizosphere of wheat and chickpea under +DR than in the −DR treatments (*P* < 0.01, Table 1).

Therefore, the presence of decaying roots with their physical, chemical and biological properties appeared to promote more similar rhizosphere microbiomes in the living roots of wheat and chickpea.

### Functional annotation

**Fig. 4:**
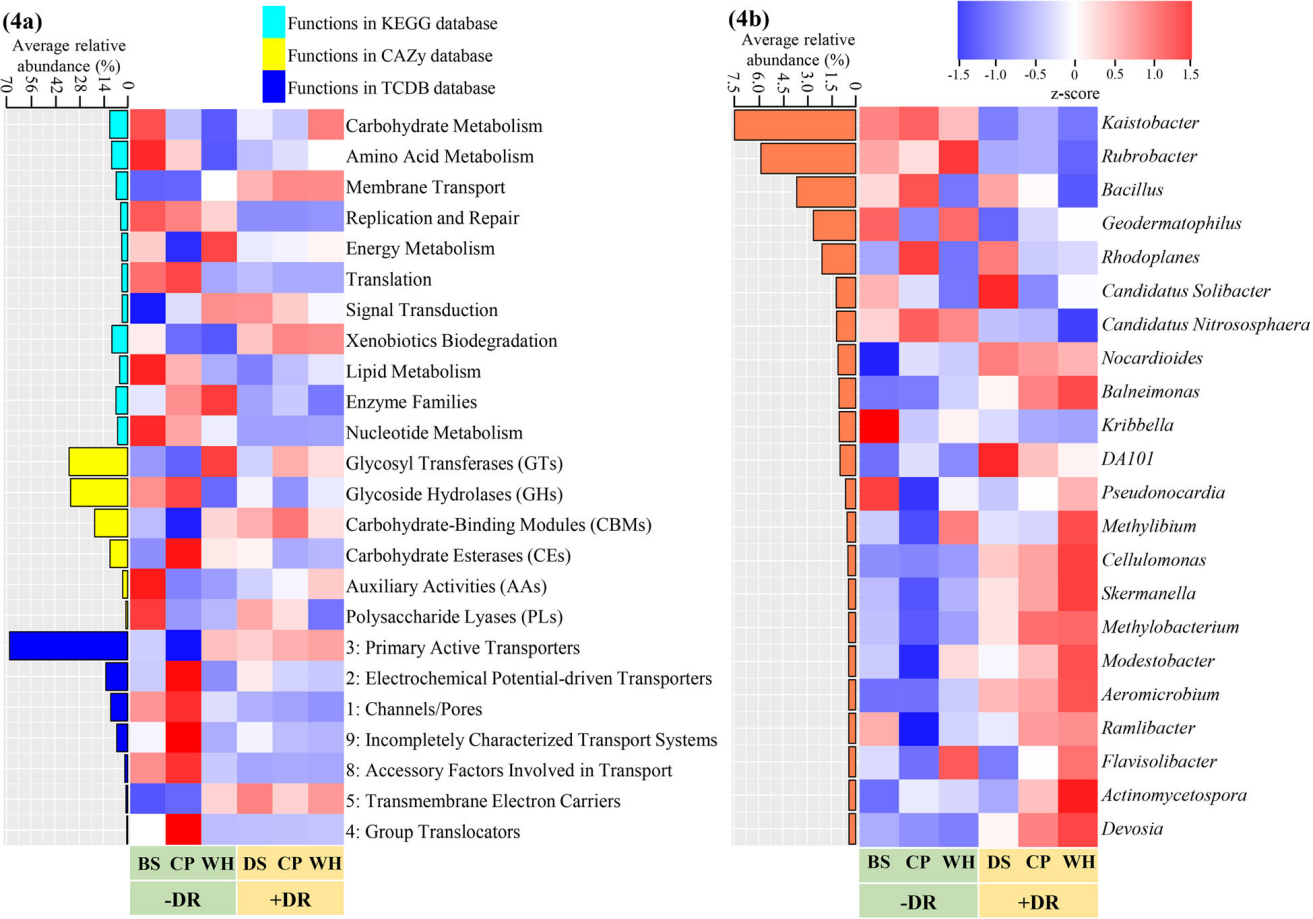
The relative abundance of microbial **a** gene groups and **b** taxonomic genus between different niches. The sampled niches included the rhizosphere of chickpea (CP) and wheat (WH) growing under + and − decaying root (DR), bulk soil (BS) sampled from the unplanted control in −DR and detritusphere soil (DS) sampled from the soil surrounding the decaying root from unplanted control in +DR. Only the groups/genus with top average relative abundance, and significantly influenced by niches (*P* value adjusted by Benjamini-Hochberg FDR < 0.05), are presented here. The mean values and standard error in the heatmap are presented in Additional file 2 Table S8 and Table S9

The relative abundance of sequenced reads grouped into microbial functional and taxonomic families was compared between different rhizospheres and bulk soil (Fig. 4). Statistical differences among niches were identified based on adjusted *P* < 0.05 by Benjamini-Hochberg FDR method in ANOVA with 3 replicates. Functional and taxonomic groups significantly responding to different niches and having the highest abundance are presented in Fig. 4.

Niches under +DR had a similar abundance of gene functions with a higher proportion of sequences belonging to ‘carbohydrate metabolism’, ‘xenobiotics biodegradation’ and ‘membrane transport’ than the plant rhizosphere niches under −DR (adjusted *P* < 0.05, Fig. 4a). Genes assigned to genetic information processing such as ‘replication and repair’ and ‘nucleotide metabolism’ in the rhizosphere microbiome were upregulated by −DR rhizobiome than by +DR (adjusted *P* < 0.05).

Under −DR, there were a similar amount of genes for ‘carbohydrate metabolism’ between the chickpea and the wheat rhizosphere microbiome, while the chickpea rhizosphere was more abundant in functions associated with carbohydrate degradation (glycoside hydrolases (GHs)) and had less abundance related to carbohydrate synthesis (glycosyl transferases (GTs)) compared to −DR wheat (adjusted *P* < 0.05). In addition, −DR chickpea rhizosphere had the lowest proportion of functions related to ‘membrane transport’ and ‘energy metabolism’, which was associated with its lowest abundance of ‘primary active transporters’.

The number of enriched or depleted genes (relative to bulk soil) was also annotated into different functional and taxonomic groups, and the enrichment of the number of functional genes among niches matched the results from the relative abundance analysis (Additional file 1: Fig. S5a-c). There were more enriched genes related to the functions of ‘membrane transport’ and ‘carbohydrate degradation’, and bacteria order Rhizobiales in plant rhizospheres under +DR than −DR.

### Taxonomic profile of niches

OTU analysis based on amplicon sequencing of 16S rRNA genes showed that the two most abundant bacteria genera *Kaistobacter* and *Rubrobacter* were more enriched without DR than with DR (adjusted *P* < 0.05, Fig. 4b). Chickpea rhizosphere without DR harboured a greater abundance of *Bacillus* than other rhizosphere niches under −DR or +DR. By contrast, the abundance of *Nocardioides*, *Cellulomonas*, *Skermanella*, *Methylobacterium*, *Modestobacter* and *Aeromicrobium* was higher with DR (0.76%) than without DR (0.34%, adjusted *P* < 0.05).

When the enriched genes of rhizosphere niches were annotated into taxonomic groups compared with bulk soil (Additional file 1: Figure S5d), the proportion of rhizosphere-enriched genes belonging to Rhizobiales (the most abundant order) was higher with +DR, compared with −DR, while the order Rhizobiales did not include the most abundant genera such as *Kaistobacter*, *Rubrobacter* or *Bacillus*.

In addition to analysing the taxonomic composition of the entire gene catalogue outlined above, the taxonomic composition of genes involved in specific functional groups was also evaluated. Non-redundant genes within each of the 8 functional groups were used for taxonomic annotation. Compared with the whole gene catalogue, Rhizobiales contributed more to the functions associated with ‘membrane transport’ (Additional file 1: Figure S6a), and Sphingomonadales contributed more to ‘transcription’ and ‘energy metabolism’. The group ‘xenobiotic biodegradation and metabolism’ involved a higher proportion of unclassified Proteobacteria. Also, the influence of experimental treatments such as decaying root and plant types on microorganism composition at order level was consistent across the tested functional groups and the whole gene catalogue, based on the Mantel analysis (Additional file 1: Figure S6b).

Two approaches to analyse microbiome taxonomic composition, amplicon sequencing on 16S rRNA genes and metagenomics sequencing, were compared. At the genus level, sequencing 16S rRNA genes lead to more sequences mapped to the identified genera than sequencing the metagenome, 39% vs 7.5% (Additional file 1: Figure S7a). There was a greater diversity of identified genera by metagenomics approach (Additional file 1: Fig. S7b). For the highly abundant bacteria genera *Kaistobacter* and *Rubrobacter*, the relative abundance was significantly correlated between the two methods (*n* = 18, Additional file 1: Fig. S7c-d), while for the genera *Bacillus* and *Geodermatophilus* which had an extremely low relative abundance (1.6 × 10^−4^–5.9 × 10^−6^) in metagenomics sequencing, the correlations between two approaches were not significant (Additional file 1: Fig. S7e-f).

### Chickpea symbiotic rhizobia

**Fig. 5:**
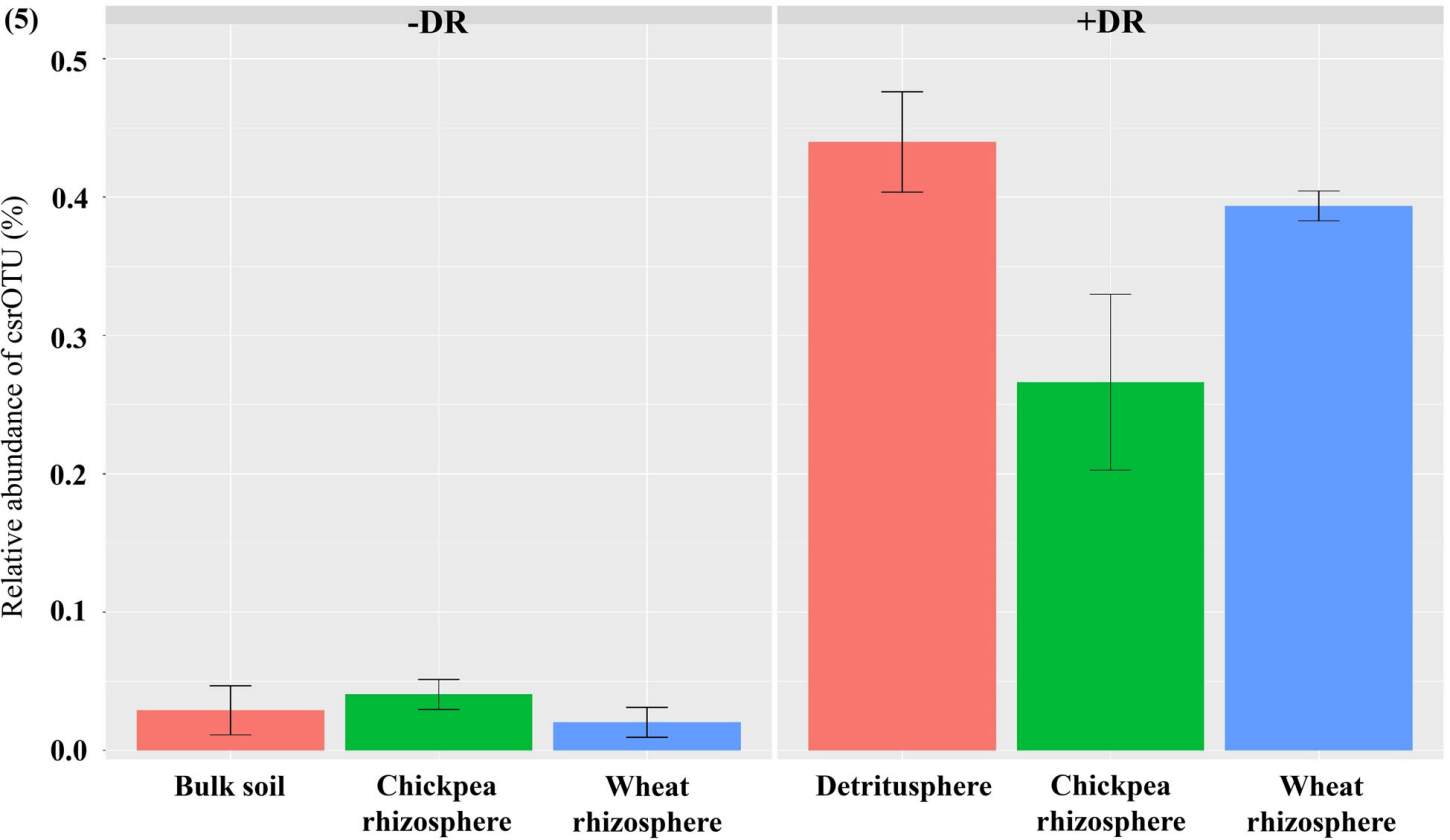
The relative abundance of chickpea symbiotic rhizobia in wheat and chickpea rhizosphere growing under + and − decaying root (DR). The chickpea symbiotic rhizobia OTU (csrOTU) was identified by analysing the 16S rRNA gene of the clean nodules. ANOVA test showed that the effect of niches was significant at *P* adjusted by Benjamini-Hochberg FDR < 0.05. Bars indicate the standard error at *P* = 0.05

Nodules were not observed in the chickpea root in the short-term soil microbiome experiment (experiment 2), due to the short growing period of 12 days. We conducted another experiment with the same chickpea cultivar in the same soil for 4 weeks, and then sequenced the 16S rRNA genes of the harvested nodule tissue. One OTU, annotated as *Mesorhizobium*, accounted for over 90% abundance in the nodule sequences was identified as the chickpea symbiotic rhizobial OTU (csrOTU). By blasting all the OTUs from the rhizosphere niches (experiment 2) against the csrOTU sequence, one OTU was confirmed as csrOTU (398/404 matching with 0 gap, Additional file 1: Fig. S8). Analysis of csrOTU in different niches showed that it was over 10 times more abundant in the niches under +DR than in –DR (*P* < 0.01). Within +DR (Fig. 5), csrOTU was less enriched in the chickpea rhizosphere than in the wheat rhizosphere and detritusphere.

### Field evaluation

A 2-year field experiment was conducted to evaluate if rhizosphere microbial assemblages in different crop types and decaying roots observed in the pot study influenced crop field performance. In the same soil, wheat and chickpea were planted with pre-cropped wheat root residues retained and removed. The results showed that the increase in grain yield in the transition from −DR to +DR was more significant for wheat than chickpea (*P* < 0.01, Additional file 1: Table S4).

### A soil microbial gene catalogue from the living-decaying rhizosphere niches

By combing all the metagenomics sequences, a non-redundant gene catalogue with 19.8 M genes, 513 bp average length and 10.16 Gbp total length was established (Additional file 1: Table S5) which had an average of 39.6% mapping rate reads (Additional file 1: Table S6). The non-redundant gene catalogue was annotated by aligning them to taxonomic and functional databases (Additional file 1: Table S7) including NCBI microbial NR database (76% genes mapped), KEGG (76% genes mapped), eggNOG (70% genes mapped for NOG+COG), CAZy (10% genes mapped) and TCDB (1.6% genes mapped).

**Fig. 6:**
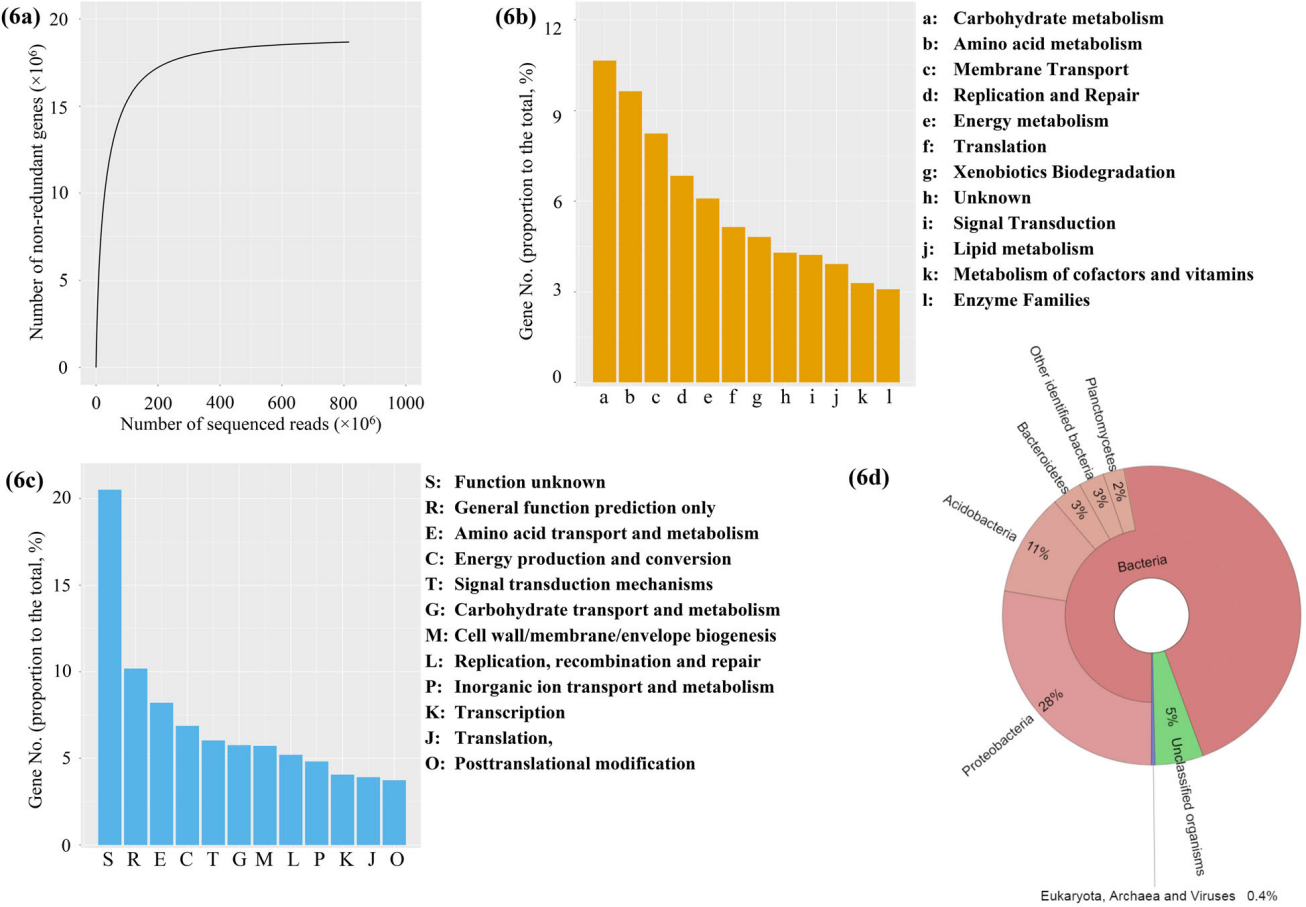
Coverage of a soil metagenome based on one pooled sample. **a** Rarefaction curves for the number of detected non-redundant genes with increased sequencing depth. The number of genes belongs to the functional groups in **b** KEGG and **c** eggNOG database and **d** taxonomic groups based on the NCBI microbial NR database using the lowest common ancestor method

As saturation was reached in rarefaction curves, the sequencing depth and established gene catalogue in the present study were considered adequate to cover the database they interrogated (NCBI Reference Sequence Database, Fig. 6a).

The proportion of genes with unknown functions was about 5% in the KEGG database, but larger in the eggNOG database at about 50% (Fig. 6b, c). The genes related to functions of membrane transport and the metabolisms of carbohydrate, amino acid, DNA and energy accounted for a majority of known functions, ~ 46% in the KEGG database (Fig. 6b) and ~ 32% in the eggNOG database (Fig. 6c).

Taxonomic classification of the gene catalogue showed that around 95% of organisms belonged to the kingdom Bacteria (Fig. 6d). Within bacteria, a large proportion (47%) of genes was detected in more than one phylum (defined as “unidentified” here). The genes belonging to a group of non-bacteria organisms (fungi, archaea and viruses) were quite minor, at less than 0.4%.

## Discussion

We present a soil microbial gene catalogue providing high coverage of the non-redundant genes, based on the niches of bulk soil, rhizosphere (living root) and detritusphere (decaying root) in an agricultural land that is representative of soils from many parts of the world (cambisols in the International World Reference Base for Soil Resources). According to the gene catalogue that we established, there was a significant interaction between crop type and cropping management on the composition and function of the rhizosphere microbiome. The detritusphere, formed by the decaying roots, modified the rhizosphere microbiome of a cereal (wheat) and a legume (chickpea) plant during the early stage of crop growth, probably due to the tight contact between the decaying roots and fine roots, and the rich and active microbiome of decaying root, capable of degrading root exudates from both cereal and legume plants (Fig. 1). A previous study demonstrated that soil microorganisms were able to utilise more carbon sourced from the decaying roots than from root exudates of a living plant, and the microbial use of residue carbon was restricted to 1 mm from the decaying root [34]. Beneficial microbial groups, e.g. *Rhizobiales* (including N_2_ fixers) and *Sphingomonadales* (root disease suppressors; 52, 53), were the the dominant groups that responded to plant type and management, especially under the influence of the detritusphere.

### Niche comparisons

The composition and functional capacity of the rhizosphere microbiome between wheat and chickpea were similar when growing in the presence of decaying roots, but heterogenous when grown in the absence of decaying roots. The possible reason for the influence of the decaying roots was that 60 to 80% of plant roots grew through existing root channels left by the previous crop, as these likely had lower resistance than the surrounding bulk soil (Fig. 1c). Thus, the detritusphere microbiome existing in the root channels of the +DR treatments determined the composition and function of rhizosphere microbiome to a greater extent than plant type did.

Furthermore, the larger number of microbes (Table 1) and their greater activity in the detritusphere (Fig. 1d) most likely degrade root exudates in the rhizosphere (Fig. 1b), and thus dilutes the influence of plant rhizosphere processes, such as root exudation, in shaping the microbiome. Similarly, it has been reported that the detritusphere stimulated enzyme activities more strongly than the rhizosphere did in roots of barley [35]. Another study using ^13^C signatures showed that carbon incorporation into microbial biomass was stronger from the decaying root than from the living root of plants up to 3 mm from the residues/roots [34].

When roots grew in the soil without decaying roots, plant root exudates drove the selection of the rhizosphere microbiome. For example, under −DR, the rhizosphere of chickpea showed a more restricted selection of microbiomes relative to wheat, as indicated by fewer microbes; a lower diversity index (Table 1); and fewer enriched/depleted genes in the chickpea rhizosphere (Fig. 2). This was correlated with the greater quantity (Fig. 2) of acidic root exudates (Table 1) detected in the chickpea rhizosphere than those in wheat.

Here, we demonstrate that the existence of decaying roots (with no change in soil chemical properties compared with −DR) can reduce the selective effects of plants on the rhizosphere microbiome structure and function, through the strong influence of the detritusphere. The capacity for young seedlings (as used in our study) to influence their rhizosphere microbiome may be restricted at such an early age when carbon allocation to growth may impede the production of root exudates. As such, our sampling of material at the early seedling stage may place important caveats on any conclusions.

No tillage, with no disturbance to the decaying root of the previous crop, is an important component of conservation agriculture (CA) that is estimated to be applied globally to 125 million hectares and has increased 3-fold during the past decade [26, 36]. Longer-term implementation of no-tillage and stubble retention practices (two components of CA) that maintain decomposing particulate organic materials from above- and below-ground plant residues (i.e. detritusphere) has been shown to affect total soil bacterial and fungal community structure (genomic community structure [17, 37, 38] and active communities (metatranscriptomics [39]) leading to biological disease suppression against soilborne pathogens [40]. Our findings here suggest that the detritusphere, shaped from the decaying root, is a key niche influencing the rhizosphere microbiome in no-tillage cropping systems. Furthermore, we did not focus on the influence of tillage on soil chemical traits such as organic C and total N, which can be changed by the long-term influence of CA [41].

The composition and function of the detritusphere microbiome might be determined by the previous crop’s rhizosphere microbiome and the quality of the root residues. Our results showed that the function and structure of the microbial community in the wheat detritusphere (soil surrounding the decaying root under +DR) was quite similar to that of wheat rhizosphere under −DR (Additional file 1: Fig. S3). The importance of dead cell wall residues in harbouring microbiota in soils has been highlighted, which accounted for around 40% of the live root-associated inhabiting bacteria in *Arabidopsis* [19]. Interestingly, wheat detritusphere microbiomes in our study were similar to wheat rhizosphere microbiomes but differed from chickpea rhizosphere microbiomes in microbial function and composition (Additional file 1: Fig. S3). This observation indicates that the microbial community formed by the rhizosphere of wheat can have a lasting impact, even when root cells become inactive. Furthermore, the high similarity of the microbiome between the wheat rhizosphere and wheat detritusphere possibly led to an improved adaptation of wheat roots to its detritusphere microsite. Our field experiment supported the finding that grain yield of wheat was more responsive to the cropping system (+/− wheat decaying root) than chickpea was (Additional file 1: Table S4).

### Taxonomic and functional annotation

The abundance of major microbial taxonomic groups is associated with their roles in agricultural production systems. For example, the present results showed that more chickpea symbiotic rhizobia enriched in rhizosphere and detritusphere under +DR than −DR (Fig. 5). More symbiotic rhizobia inhabiting the detritusphere might have a greater probability to nodulate with the host legume roots, as the legume roots grow through the detritusphere in the +DR treatment. Our findings support the previous result that retaining decaying roots through no-tillage maintained legume rhizobial populations and diversity, compared with the removal of decaying roots [42].

Moreover, a dominant bacterial genus, *Kaistobacter*, which is associated with disease suppressiveness [43] was less abundant in +DR than in −DR, which indicated a higher chance for plants to be infected by pathogen disease under +DR. *Kaistobacter* is a member of the bacterial community that responds to the availability of easily degradable C compounds, especially plant root exudates [43, 44]. Chickpea rhizosphere under −DR assembled more *Kaistobacter* than wheat (Fig. 4b), possibly due to its acidic pH and the greater presence of root exudates (Fig. 2 and Table 1).

Another dominant bacterial genus, *Rubrobacter*, was also more abundant under −DR than +DR (Fig. 4). It has been reported that *Rubrobacter* was widely distributed in Australian arid soils [45], and firstly isolated from an Australian acid soil [46]. *Rubrobacter* was physiologically active in the plant rhizosphere soil with high mineral nutrients, but low organic carbon [47].

Under the presence of a decaying root, the rhizosphere microbiome was enriched with more genes associated with the metabolism of carbohydrate (especially C degradation) and membrane transporters. This possibly relates to the influence of the detritusphere providing C and N pools and more active microbes to enhance C and N utilisation and uptake through microbial cell transporters, as indicated by the significant degradation of root exudation in the rhizosphere under +DR (Fig. 1b). Additionally, the enriched chickpea symbiotic rhizobia in +DR rhizospheres possibly contribute to the higher abundance of transporter functions in that region, as the rhizobial genome is rich in transporter genes, representing over 10% of the total annotated proteins [48].

When plants grow in the absence of decaying roots, rhizosphere microbial functions of chickpea were enriched more in carbohydrate degradation metabolism than in wheat. Greater carbohydrate degradation may be associated with stronger exudation activity from the chickpea root and its acidic root exudation compounds (Fig. 1a, b; Table 1 in the present study and [33]). Under −DR, the microbiome functions of the wheat rhizosphere were characterised by enhanced carbohydrate synthesis and its related energy metabolism compared with chickpea, possibly due to the weaker C exudation of wheat root than chickpea, as measured in the present study.

The functional relatedness between niches was independent of their taxonomic assembly, as the distance among samples in taxonomic composition was consistent across functional groups with homogeneous and heterogeneous niche effects (Additional file 1: Fig. S6b). Our results are similar to previous metagenomic analyses on the niches of the phyllosphere [49] and land use [50] and support the neutral theory [51] that the taxonomic composition of the microbial community is assembled by stochastic processes in the rhizo-detritusphere microsite environment.

### A soil microbial gene catalogue from the living-decaying rhizosphere niches

The microbial gene catalogue presented here represents multiple samples collected in an agricultural field that is a typical cropping system in a Mediterranean semi-arid climate, representative of environments that are important regions for global crop production and crop diversity hotspots [52, 53].

Our soil metagenome has higher assembly quality with greater coverage compared with other assembled soil metagenomes. For example, when compared with the standard soil metagenome of Iowa corn and native prairie soils in the USA [7], which also applied de Bruijn graph-based approach for sequence assembly, our soil metagenome has more assembled contigs (12.9 million vs 1.9 million and 3.1 million with a minimum length of 300 bp), a longer total assembly length (11 Gbp vs 0.912 Gbp and 1.5 Gbp) and a higher assembly rate (50% vs 20%) with sequenced clean data of 205 Gbp vs 140 Gbp and 252 Gbp.

To assess the sequence diversity present in a soil sample, the estimation of the coverage provided by the specific metagenome dataset and the extent of the real diversity of the community in an environment were assessed [9]. A previous estimation of the soil metagenome was 10^12^ genes per gram of soil for bacteria, based either on the number of bacteria and effective genome size [54, 55] or 50 Tbp sequence required to cover the whole metagenome of 1 g of soil [7, 56]. Our assembled gene catalogue has less genes than the previous estimations but with improved assembly quality. The possible reasons include, firstly, that our soil samples are from a cropping agroecosystem with low diversity of above-ground vegetation, with mostly 1–2 crop species present in the field each year, especially over the last 50 years. Additionally, soil organic carbon in our study is low (1.4%) compared with more diverse systems such as prairie grasslands and forest ecosystems with higher soil organic C concentrations. Consequently, agricultural land may have reduced richness of functional genes in the soil microbiome [7, 57]. Secondly, our sampling site was from a semi-arid Mediterranean climate with extremely dry and hot summers for > 4 months per year. Soils from global drylands have demonstrated that diversity and abundance of microorganisms are reduced with increasing aridity [58]. Finally, microbial genomes might share the same, or similar genes, when adapted to the same environment. Horizontal gene transfer can enrich the proportion of shared genes [59], and the percentage of shared genes rises with the inclusion of more genomes [60]. In more disturbed systems, e.g. in our cropping soil with low organic matter and microbial number and diversity, the annual disturbance would potentially facilitate the increased interactions between microsites.

Bacteria were the dominant microbial taxa in the gene catalogue and in soil samples, demonstrated by their dominance in gene number (~ 95%) and higher copy number. While the fungal communities were quite minor (gene number < 0.4% of the gene catalogue, and copy number only 1/100–1/200 of the bacterial community), this is possibly due to the environment where our soils were collected. Previous studies on the global fungal diversity showed that annual rainfall was the best predictor of fungal richness with a positive effect, and South Australian soil had a low level of fungal diversity [61].

## Conclusions

In this study, we established a microbial gene catalogue for a long-term agricultural soil from a Mediterranean-type climate. We demonstrated the important role that the detritusphere (formed by decaying root) plays in determining the rhizosphere microbiome structure and potential function. The existence of the decaying root homogenised the rhizosphere microbiome of different crops, through close root contact of the decaying root with fine living root, and the development of an active microbiome that degrades plant root exudates. These results demonstrate that under conservation agriculture systems, microsites such as detritusphere and rhizosphere are in constant interaction, providing microbial hotspots moderated by the crop type and physico-chemical properties of the system.

The foundation of crop selection is based on bio-environment (soil type, aspect and climate), market opportunities, crop history (rotation) and management (tillage and nutrition). Underpinning these holistic factors is the emerging awareness that the soil can also be considered in a more reductionist way, particularly pertaining to the functioning of the soil microbiome. Our study examined the basis of a soil microbiome in typical cropping soils including functional and taxonomic diversity, to understand the opportunities that exist for managing these microbiomes. This greater understanding of the soil microbiome can lead to better management of the soil resource and sustainable crop production.

Our findings illustrate the role that the microbiome plays at the functional level. Indeed, it may now be possible to manage and stabilise within any preferred crop rotation functions such as carbon decomposition, nutrient cycling and disease suppression through the selection of crops and management practices. However, the results also show that the microbiome is extremely dynamic. In particular, it will be valuable to compare the microbiome composition of other soils from comparable and contrasting dryland environments, to identify common edaphic and environmental drivers and to establish any underlying common compositional structures.

## Methods

### General experimental design

The intact soil cores with decaying root (+DR) and the soils for repacked columns representing the removal of decaying roots through tillage (−DR) were collected from the same field. Wheat (*Triticum aestivum* cv. Justica CL Plus), chickpea (*Cicer arietinum* cv. Hattrick) and unplanted controls were grown under +DR and −DR conditions in a controlled environment with three replicates. Using the same plant-soil design and growing system, 4 individual microcosm experiments were conducted to evaluate the plant root development, rhizosphere microbiome assemblages, rhizosphere metabolism and soil microbial respiration.

### Field site and soil collection

Soil was collected from a farming system at the Roseworthy Campus (34° 53′ S, 138° 724′ E) of The University of Adelaide, Australia. Roseworthy is a typical dryland cropping area with a Mediterranean-type climate: 463 mm annual rainfall (334 mm in the April to October growing season), mean maximum temperature of 22.5 °C and mean minimum temperature of 10.0 °C over the last 50 years [62]. The soil at the sample area is a brown Earth soil (hypercalcic, red, chromosol [63]). Soils with wheat stubble residues were sampled in January 2015 with less than 10 mm rainfall in the previous 40 days. The cropping history of the sampled site was in accordance with the local farmers’ current practice: one season legume crop followed by 3–4 cereal crops. The detailed rotation was lentil (*Lens culinaris*) in 2012 and wheat (*Triticum aestivum* cv. Justica CL Plus) in 2013 and 2014. The sample site was previously managed under no-tillage and stubble retention practices for more than 15 years. Other management practices such as fertilisation, herbicide/fungicide application and row spacing at seeding followed local farmer practices.

Intact soil cores were collected from a 50 m × 200 m area in the middle of the 20-ha field, with an even distribution between sample sites. Sterilised PVC tubes (50 mm diameter × 100 mm long) were pushed gently into the soil with a hydraulic probe, ensuring that there were 4 stems of wheat stubble residues from the previous crop in the centre of each tube. The aboveground stubble was cut off after sampling. For the −DR treatment, simulating conventional tillage effects, the intact soil cores were firstly cracked to remove the root residues by sieving (5 mm) and then mixed and repacked into PVC tubes with the same bulk density (1.48 g cm^−3^). The other soil cores were kept intact as the +DR treatment. Soil analysis for physio-chemical properties tested both + and −DR using the protocol from Rayment and Lyons [64].

PVC tubes were transferred to a controlled environment growth chamber (12 h light, 800 μmol m^−2^ s^−1^ at 20 °C and 12 h darkness at 10 °C) within 2 h of collection. In these experiments, wheat (cultivar Justica CL Plus) and chickpea (*Cicer arietinum* cv. Hattrick) represented cereal and legume crops, respectively, with contrasting root exudation profiles [33]. Surface-sterilised seeds were germinated in petri dishes first and then planted into the soils at 1 cm depth after the first root had emerged to 5 mm. Unplanted tubes of + and −DR were used as controls. All tubes were weighed every day and irrigated by autoclaved water as necessary to keep the soil moisture content at 80% of field capacity.

### Experimentation

#### Controlled environment experiments

##### Experiment 1: Root development

The experiment was arranged as a completely randomised design with 3 factors (decaying root: + and −; plant type: wheat, chickpea and unplanted; and harvest timing) and 4 replications. Replicated tubes of each treatment were harvested at 3, 6, 8, 10 and 12 days after sowing, to evaluate the root development relative to the stubble residue roots. The experimental duration was short (12 days) because (1) the active roots of young seedlings more accurately reflect the genetic variation in root exudation, and its relationship with the establishment of rhizosphere microbiome, and (2) early harvest can reduce the impacts such as uptake of immobile nutrients and root tissue decay, which may affect the rhizosphere microbiota formation between different crop species.

After cutting off the plant shoot, the tube was soaked in water at 4 °C for 24 h to disperse most of the root-attached soil. Subsequently, the roots were washed free of soil using a high-pressure shower (Additional file 1: Fig. S1). The stubble residue roots and plant fresh roots were separated to analyse for root length, using the WinRHIZO system (Regent Instruments, Inc., Quebec City, QC, Canada) with a scanner (Epson Expression 10000XL, Epson Inc., CA, USA). Root length density was calculated based on the total root length divided by the tube volume. Using the same scanned image, root distortion, which reflects changed root direction from the original trajectory due to physical impediment (Additional file 1: Fig. S1), was estimated using the method from Zhou et al. [29].

At 12 DAS, when the root length density of sown wheat seedlings was similar to that of the stubble residue root (Additional file 1: Fig. S9), the sampling for root channel occupation was initiated. Root channel occupation is defined here as the proportion of the number of newly grown roots inside the antecedent stubble residue root channel. This was evaluated using a modified method from White and Kirkegaard [28]. Firstly, the intact soil core under +DR treatments was transversely cut into 3 sections at 4, 6 and 8 cm depth of the profile. On the horizontal surface of each section, the total number of fresh roots and the number of fresh roots occupying the residue root channel were recorded under a stereo microscope. Roots on the tube wall were not considered in calculations.

##### Experiment 2: Rhizosphere microbiome

Using the same growing condition to experiment 1, experiment 2 was arranged as a completely randomised design with 2 factors (decaying root: + and −; plant type: wheat, chickpea and unplanted) and 3 replications. Rhizosphere soils were sampled at 12 DAS, following a protocol modified from that of Bulgarelli et al. [19]. Firstly, after discarding the roots on the tube wall and bottom, plants were shaken by hand to further remove bulk soil. The roots, which had ~ 1 mm soil attached, were transferred into 50-mL Falcon tube with 15-mL sterile PBS buffer. The Falcon tube was located on an orbital shaker for 20 min at 180 rpm. Roots were removed using sterilised tweezers, and the soil suspension was passed through a 0.5-mm sieve to remove any root residue. Finally, the resulting soil suspension was centrifuged at 2000 rpm for 20 min. After removing the supernatant, the pellet was defined as rhizosphere soil. The bulk soil in the unplanted −DR and soil surrounding the decaying root in unplanted +DR (detritusphere) were collected using the same procedure as for the controls. The total DNA for the 18 samples (Additional file 1: Table S1) were extracted using a PowerSoil DNA isolation kit (Mo Bio, Carlsbad, CA, USA) based on the manufacturer’s instructions.

##### Experiment 3: Root exudation

A similar tube experiment was conducted to measure the root exudates from the plant rhizosphere. The changes of root exudate abundance between sterilised and non-sterilised condition were determined to indicate the exudate degradation by rhizosphere microbial activity. Experiment 3 was arranged as a completely randomised design with 3 factors (decaying root: + and −; plant type: wheat, chickpea and unplanted; and sterilisation: + and −) and 5 replications. PVC tubes with intact and repacked soils were sterilised by 25k Gray gamma radiation, and another group of tubes was the non-sterilised control. Wheat, chickpea and unplanted controls were established in both sterilised and non-sterilised tubes under the same environment and management as before. A soil-based approach was applied to sample root exudate [65]. The attached soil on live or decayed root sections was washed into 50-mL Falcon tube with 10-mL deionised water and then microwaved by a 700-W microwave oven for 10 s to prevent metabolic activities. Samples were washed on an orbital shaker for 30 min at 180 rpm. Roots were discarded. Soil suspensions were transferred through a filter paper (Whatman no. 42). The collected soil was dried at 105 °C to measure the dry weight. The filtered solution was transferred through a syringe filter (0.1 μm) and then stored in − 80 °C freezer for further analysis. Carbohydrates and amino acid compounds were analysed by Centre for Carbon, Water and Food in The University of Sydney, following the method in [66]. The measured compounds were averaged with the amount of dry soil and calibrated by the bulk soil from unplanted −DR and unplanted +DR to measure root exudates. Meanwhile, the same rhizosphere soils collected from both sterilised and non-sterilised conditions were analysed for pH.

##### Experiment 4: Soil microbial respiration

Five replicates of unplanted tubes with and without DR were used to study the soil respiration. All tubes were sealed at the bottom to keep the moisture content at 80% field capacity and then transferred to 1 L glass jars with sealed lids. Soil respiration was determined by measuring the CO_2_ concentration in the headspace of the jars using an infrared analyser (Model 1450, Servomex Group, Crowborough, UK [67]). After each measurement, jars were vented using a fan to refresh the headspace. The amount of CO_2_ accumulated during this interval was used to indicate soil respiration. Linear regression based on the injection of known amounts of CO_2_ into empty jars of the same size was used to calibrate the relationship between CO_2_ concentration and detector reading.

##### Chickpea symbiotic rhizobia identification

To identify the 16S rRNA gene sequence of chickpea symbiotic rhizobia and analyse its abundance in different niches, the same chickpea cultivar was grown in 3 pots for 4 weeks using the soil with decaying residues removed as per experiments 1 to 4. Three nodules of each pot were harvested, and the attached soil was removed as described for root cleaning in experiment 2. The DNA of cleaned nodules were extracted, amplified for 16S rRNA genes and sequenced using the same methods as experiment 2.

#### Field experiment

To compare grain yield production of wheat and chickpea under different cropping management, a 2-year field experiment on the same agricultural land as soil collected was performed with two types of decaying root management: DR removed by tillage and DR retained with no tillage, two crops (wheat and chickpea), and 9 replicates of a paired samples in a *t* test design. The detailed management of the field trial is reported in Kitonyo et al. [68]. In each year, plants were sown into the field with pre-cropped wheat residue. Grain yield was harvested at maturity.

## Data analysis

### Metagenome sequencing and bioinformatics

The bioinformatic analysis of the DNA sequence in our study was modified from the guideline of Quince et al. [69]. The summarised flowchart is presented in Additional file 1: Fig. S10. Library construction, DNA sequencing and quality control (QC) of the sequence were performed by BGI (Shenzhen, China) based on the HiSeq 2000 platform according to the manufacturer’s instructions (Illumina, San Diego, CA, USA). The sequencing was conducted under 100-bp pair-end runs. The sequenced reads were removed if they met any one of the following criteria: (1) with ≥ 20% low-quality bases, (2) with adapter contamination (15 bases overlapped by reads and adapter with maximal 3 base mismatches allowed), (3) with N base (also removing paired reads) and (4) with low complexity (reads with 10 consecutive reads of the same base).

All clean reads of the 18 samples were pooled together for metagenomic de novo assembly rather than the assembly of each sample independently following the method from [8]. This was done (1) since all of our samples are soils from different niches from the same farm, which may have high similarity among microbial communities; (2) to establish a soil microbial gene catalogue covering all the niches of one particular farm including bulk soil, rhizosphere and detritusphere; and (3) to provide more information through pooling samples [70]. Single-sample assembly of 6 samples was also performed, but the assembly rate (percentage of reads able to map the assembled contigs) was much lower than the mix-sample assembly (9–25% vs 50.7%).

MEGAHIT [71] based on a succinct *de Bruijn* graph [72] was used to assemble all the clean reads with the “meta-large” parameters set up in the software. To evaluate the quality of metagenomic assembly, we calculated the total number, total length, N50 and average length of the assembled contigs. Also, all the clean reads were mapped to the assembled contigs to generate the assembly rate for each sample using SoapAligner software with 90% identity [73].

Using the assembled contigs with a length of > 300 bp, gene prediction was carried out on the MetaGeneMark software [74] with default parameters. Also, the number, total length, average length and GC content of predicted genes were calculated.

Using the CD-HIT software with 95% sequence identity [75], the redundant predicted metagenomic genes were removed to generate the non-redundant gene catalogue. To generate the gene abundance, the clean reads for each sample were aligned to the non-redundant genes using SoapAligner software [69] with 90% identity. The relative abundance of genes in each sample was adjusted by gene length, as reads have a higher chance to hit the longer genes [1].

The non-redundant protein sequences were aligned against NCBI microbial NR database (Bacteria, Fungi, Archaea and Virus) and four functional databases including KEGG [76], eggNOG [77], CAZy [78] and TCDB [79] using BLAST software with an *e* value ≤ 1e−5. The taxonomic annotation for each gene was carried out using the lowest common ancestor (LCA) method [1] applied by MEGAN [80]. Blast coverage ratio (BCR, the percentage of aligned length between query and reference) was selected with a cut-off of ≥ 40%. Based on the lowest blast *e* value, the functional information of the best aligned hit was selected as the metagenomic gene functional annotation [1]. The functional gene abundance profile was generated using non-redundant gene catalogue and abundance.

In the functional database, orthologous genes were classified into one catalogue and named as a KEGG orthologous group (KO).

### 16S rRNA gene sequencing and bioinformatics

Using the same extracted DNA, the V3–V4 regions of 16S rRNA genes were amplified by the forward primer 341F (CCTAYGGGRBGCASCAG) and reverse primer 806R (GGACTACNNGGGTATCTAAT). PCR products were sequenced on an Illumina MiSeq platform with 300-bp paired-end reads. The flowchart for bioinformatics analysis of the 16S rRNA gene sequence is shown in Additional file 1: Fig. S10. Raw reads were processed for QC using the method as described above for metagenome sequencing. The clean reads were passed to the open reference operational taxonomic unit (OTU) picking and assignment pipeline as described by Bissett et al. [81]. The codes and detailed method can be found from the link http://www.bioplatforms.com/wp-content/uploads/OTU_pipelines.pdf. Simply, sequences with ≥ 97% similarity were clustered as one OTU using UPARSE [82]. To obtain OTU abundance, clean reads were mapped back to OTUs with a minimum identity of 97% by USEARCH (version 8.0.1623) [83]. OTUs were annotated for taxonomies using the Greengenes database (vs 13.8) [84] and Wang classifier [85] in MOTHUR [86] at 60% sequence similarity cut-off.

The same bioinformatics was applied to the 16S rRNA gene sequence of the chickpea nodule tissue. One OTU with over 90% abundance in the nodule was identified as the chickpea symbiotic rhizobial OTU (csrOTU). All the OTUs assembled from the rhizosphere and bulk soil were blasted against the csrOTU using ViroBLAST [87]. The abundance of one matched OTU was used to indicate the proportion of chickpea symbiotic rhizobia at different niches.

The quantity of microbial DNA in the samples was measured by RT-qPCR using the Femto™ Bacterial and Fungal DNA Quantification Kit (Zymo Research, USA) with primers targeting the 16S rRNA gene and internal transcribed spacer (ITS) gene. Three technical replicates per sample were analysed. According to the protocol from the kit manual, the concentration of bacteria and fungi in the samples was converted into gene copy number per unit dry soil calibrated with the standards of *Escherichia coli* strain JM109 and *Saccharomyces cerevisiae* strain TMY18.

### Statistics

Non-redundant gene was firstly used as the basic unit for our statistical analysis. Differentially abundant genes were identified using a generalised linear model (GLM) from edgeR [88, 89]. Firstly, low abundant genes were filtered out based on the criteria of at least 3 samples with over 5 reads. A trimmed mean of *M* values (TMM) method [89] was applied to normalise the library size across all the samples. The fitness of the negative binomial model was tested by estimating the biological coefficient of variation before the analysis was carried out. Differential abundance for each gene was determined by the GLM likelihood ratio test with the estimated dispersions using the Cox-Reid profile-adjusted likelihood method. Rather than test for genes that have log-fold-changes different from 0 compared with the control, we used *glmTreat* to test whether the log2-fold-change is greater than 1 (whether the treatment is statistically twice more than the control). The bulk soil treatment was considered as the control to compare with each of the other treatments for detecting differentially abundant (enriched/depleted) gene with the FDR adjusted *P* value < 0.01.

The identified differentially abundant genes were assigned into different databases including the NCBI microbial NR database, KEGG, CAZy and TCDB to illustrate the proportional distribution profile of gene number. Similar differential abundance analysis using KOs and OTUs as the basic unit was also performed.

Besides differential abundance analysis, the relative abundance of sequenced reads grouped into microbial functional and taxonomic families was compared between different rhizospheres and bulk soil (Additional file 1: Table S1) using the Statistical Analysis of Metagenomic Profiles (STAMP) package [90] and Benjamini-Hochberg FDR method to correct for the *P* value.

Multivariate analysis was conducted for non-redundant genes, OTUs and root exudate compounds. Unconstrained principal coordinate analyses (PCoA) based on Bray-Curtis distance were calculated by the pcoa() function from the R Package Vegan [91]. Permutational MANOVA with a maximum of 999 permutations using the adonis() function was conducted to test the effect of experimental factors on β-diversity. Alpha-diversity indices for each sample were estimated also using R Package Vegan based on rarefied OTUs and genes table.

Eight major functional groups in KEGG pathways were analysed for the function-taxonomy relationship. All non-redundant genes within each of the 8 functional groups were aligned for taxonomic annotation, and then the taxonomic data were analysed as described above.

Individual-based rarefaction analysis was conducted for non-redundant gene catalogues using the R package rareNMtests. All the sequences were re-sampled by randomisation to calculate the richness of non-redundant genes in the subsample.

One-way ANOVA was conducted to test the effect of experimental factors on soil physiochemical properties, root morphological traits, soil respiration, shoot biomass ratio, root exudate compound concentrations and alpha-diversity indices using GenStat vs15 (VSN International Ltd., Hertfordshire, UK).

## Supplementary information

**Additional file 1: Table S1.** The experimental design. **Table S2.** Physical and chemical properties of soils. **Table S3.** MANOVA table. **Table S4.** The grain yield production influenced by decaying roots of pre-crop in the field experiment. **Table S5.** Statistical results of the assembled metagenome. **Table S6.** Statistical summary of the metagenomics sequencing and assembly. **Table S7.** The number of genes and reads aligned to the non-redundant gene catalogue and the annotated databases. **Figure S1.** Pictures of fresh root-residue root contact and root distortion of wheat and chickpea. **Figure S2.** Root distortion rate of wheat and chickpea influenced by decaying root. **Figure S3.** Comparing the functional and taxonomic structure of rhizosphere microbiome between wheat and chickpea growing under + and – decaying root. **Figure S4.** Comparison of differentially abundant genes between rhizosphere and detritusphere microbiome under + and – decaying root. **Figure S5.** Functional and taxonomic annotation of the differentially abundant genes in the rhizosphere of wheat and chickpea under + and – decaying root. **Figure S6.** Taxonomic structure of rhizosphere microbiome at different metabolic pathways. **Figure S7.** Comparison of two approaches, amplicon sequencing on 16S rRNA genes and metagenomics sequencing, to analyse microbiome taxonomic composition at genus level. **Figure S8.** Identification of chickpea symbiotic rhizobia. **Figure S9.** Root length density of wheat and chickpea under + and – decaying root (DR) changed with days after planting. **Figure S10.** Flowchart of bioinformatics analysis for metagenomics sequencing and 16S rRNA gene sequencing.

**Additional file 2: Table S8** and **Table S9**. The relative abundance of microbial gene groups and taxonomic genus between different niches. This file includes the mean values and standard error for the heatmap in Fig. 4a and Fig. 4b.

**Additional file 3:** Review history.

## Data Availability

Sequenced metagenomic clean data during the current study were submitted to the European Nucleotide Archive (http://www.ebi.ac.uk/ena/) with the study accession number PRJEB30524 [92]. The amplicon sequences on 16S rRNA genes were uploaded to Sequence Read Archive of The National Center for Biotechnology Information (https://www.ncbi.nlm.nih.gov/sra) with BioProject ID: PRJNA609629 [93]. The original data and source code for data analysis are available in the GitHub repository licenced under GNU GPLv3 https://github.com/DentonLab/decaying-root-microbiome [94] and deposited in Zenodo with DOI 10.5281/zenodo.3692465 [95].
