# Supplementary material for: The preceding root system drives the composition and function of the rhizosphere microbiome
Source: Genome Biol. 2020 Apr 6;21:89. doi: 10.1186/s13059-020-01999-0 (PMC7137527; doi:10.1186/s13059-020-01999-0)
Supplement: Supplementary file 1 — Additional file 1: Table S1. The experimental design. Table S2. Physical and chemical properties of soils. Table S3. MANOVA table. Table S4. The grain yield production influenced by decaying roots of pre-crop in the field experiment. Table S5. Statistical results of the assembled metagenome. Table S6. Statistical summary of the metagenomics sequencing and assembly. Table S7. The number of genes and reads aligned to the non-redundant gene catalogue and the annotated databases. Figure S1. Pictures of fresh root-residue root contact and root distortion of wheat and chickpea. Figure S2. Root distortion rate of wheat and chickpea influenced by decaying root. Figure S3. Comparing the functional and taxonomic structure of rhizosphere microbiome between wheat and chickpea growing under + and – decaying root. Figure S4. Comparison of differentially abundant genes between rhizosphere and detritusphere microbiome under + and – decaying root. Figure S5. Functional and taxonomic annotation of the differentially abundant genes in the rhizosphere of wheat and chickpea under + and – decaying root. Figure S6. Taxonomic structure of rhizosphere microbiome at different metabolic pathways. Figure S7. Comparison of two approaches, amplicon sequencing on 16S rRNA genes and metagenomics sequencing, to analyse microbiome taxonomic composition at genus level. Figure S8. Identification of chickpea symbiotic rhizobia. Figure S9. Root length density of wheat and chickpea under + and – decaying root (DR) changed with days after planting. Figure S10. Flowchart of bioinformatics analysis for metagenomics sequencing and 16S rRNA gene sequencing. [file 13059_2020_1999_MOESM1_ESM.pdf]

Additional file 1

Table S1. 2
Table S2. 3
Table S3. 4
Table S4 5
Table S5. 6
Table S6. 7
Table S7. 8
Fig S1. Pictures of fresh root-residue root contact and root distortion of wheat and chickpea under + and – decaying root (DR). 9
Fig S2. Root distortion rate of wheat and chickpea influenced by decaying root (DR). 10
Fig S3. Comparing the functional and taxonomic structure of rhizosphere microbiome between wheat and chickpea growing under + and – decaying root (DR). 11
Fig S4. Comparison of differentially abundant genes between rhizosphere and detritusphere microbiome under + and – decaying root (DR). 12
Fig S5. Functional and taxonomic annotation of the differentially abundant genes in the rhizosphere of wheat and chickpea under + and – decaying root (DR). 14
Fig S6. Taxonomic structure of rhizosphere microbiome at different metabolic pathways. ... 15
Fig S7. Comparison of two approaches, amplicon sequencing on 16s rRNA genes and metagenomics sequencing, to analyse microbiome taxonomic composition at genus level. .. 17
Fig S8. Identification of chickpea symbiotic rhizobia 18
Fig S9. Root length density of wheat and chickpea under + and – decaying root (DR) changed with days after planting. 19
Fig S10. Flowchart of bioinformatics analysis for metagenomics sequencing and 16S rRNA gene sequencing 20

Table S1. The experimental design and sample collection of present study

Sample ID	Decaying root	Plant Type	Replicate	Sampled Niche
A1A	−	Unplanted	1	Bulk soil
A2A	−	Unplanted	2	Bulk soil
A3A	−	Unplanted	3	Bulk soil
B1A	+	Wheat	1	Wheat rhizosphere (living root)
B2A	+	Wheat	2	Wheat rhizosphere (living root)
B3A	+	Wheat	3	Wheat rhizosphere (living root)
C1A	−	Wheat	1	Wheat rhizosphere (living root)
C2A	−	Wheat	2	Wheat rhizosphere (living root)
C3A	−	Wheat	3	Wheat rhizosphere (living root)
D1A	−	Chickpea	1	Chickpea rhizosphere (living root)
D2A	−	Chickpea	2	Chickpea rhizosphere (living root)
D3A	−	Chickpea	3	Chickpea rhizosphere (living root)
E1A	+	Chickpea	1	Chickpea rhizosphere (living root)
E2A	+	Chickpea	2	Chickpea rhizosphere (living root)
E3A	+	Chickpea	3	Chickpea rhizosphere (living root)
F1A	+	Unplanted	1	Detritusphere (decaying root)
F2A	+	Unplanted	2	Detritusphere (decaying root)
F3A	+	Unplanted	3	Detritusphere (decaying root)

Table S2. Physical and chemical properties of soils under the + and – decaying roots (DR). The intact soil cores with root residue collected from the field were used as +DR. –DR was generated by cracking the intact soil cores, removing root residues and repacking into tubes with the same bulk density with +DR. Soil samples for the analysis exclude the root residues in +DR. The symbol * indicates the significant difference of ANOVA test between + and – DR at P-value < 0.05. ns means not significantly difference.

	+DR	-DR	
Bulk density, g cm^{-3}	1.48	1.48	ns
Field capacity, %	26.98±2.74	25.97±0.07	ns
Clay, %	24.79±2.31	24.89±1.99	ns
Sand, %	69.86±2.47	67.67±2.44	ns
Ammonium Nitrogen, mg kg^{-1}	3.07±0.01	3.58±0.51	*
Nitrate Nitrogen, mg kg^{-1}	11.24±1.02	12.79±2.63	ns
Phosphorus Colwell, mg kg^{-1}	72.75±0.38	75.12±2.57	ns
Potassium Colwell, mg kg^{-1}	727.66±15.78	714.95±9.81	ns
Sulphur, mg kg^{-1}	6.29±0.05	7.13±0.53	*
Organic Carbon, %	1.40±0.06	1.43±0.12	ns
Conductivity, dS m^{-1}	0.09±0.01	0.10±0.01	ns
pH ($CaCl_2$)	6.25±0.15	6.15±0.05	ns
pH (H_2O)	7.05±0.05	6.95±0.15	ns
DTPA Copper, mg kg^{-1}	1.27±0.01	1.33±0.08	ns
DTPA Iron, mg kg^{-1}	25.92±1.23	27.87±4.58	ns
DTPA Manganese, mg kg^{-1}	10.95±0.35	12.07±2.32	*
DTPA Zinc, mg kg^{-1}	1.38±0.13	1.55±0.13	ns
Exc. Aluminium, cmol kg^{-1}	0.10±0.01	0.08±0.01	ns
Exc. Calcium, cmol kg^{-1}	10.12±0.56	10.64±0.54	ns
Exc. Magnesium, cmol kg^{-1}	2.86±0.24	2.94±0.10	ns
Exc. Potassium, cmol kg^{-1}	1.55±0.24	1.70±0.02	ns
Exc. Sodium, cmol kg^{-1}	0.34±0.03	0.30±0.04	ns
Boron (hot $CaCl_2$), mg kg^{-1}	2.05±0.04	1.98±0.08	ns
Total Nitrogen, %	0.16±0.01	0.16±0.01	ns
Total Carbon, %	1.62±0.11	1.71±0.12	ns
C:N	10.26±1.01	10.76±0.43	ns

Table S3. Multivariate analysis of variances (MANOVA) table using the normalised OTU and gene counts. MANOVA was based on Bray–Curtis distance and maximum 999 permutations. Note: PT – plant type; DR – the existing of decaying root

		Degree of freedom	Sums of Squares	Mean Squares	F Model	R^2	P value
OTUs	PT	1	0.0127	0.0127	3.0718	0.1352	<0.05
	DR	1	0.0388	0.0388	9.3727	0.4124	<0.01
	PTxDR	1	0.0095	0.0095	2.2820	0.1004	0.065
Genes	PT	1	0.1416	0.1416	6.0832	0.1631	<0.01
	DR	1	0.4136	0.4136	17.767	0.4763	<0.01
	PTxDR	1	0.1268	0.1268	5.4489	0.1461	<0.01

Table S4. The differentiation of grain yield production between decaying roots of pre-crop retained (+DR) and removed (-DR) in the field experiment. Data was presented as mean±standard error. t-test was conducted to compare +DR/-DR ratio for chickpea and wheat, respectively. * and ** indicated significant different at $P < 0.05$ and $P < 0.01$, respectively.

	Grain yield (t ha^{-1})						
	Chickpea			Wheat			t-test
Year	-DR	+DR	+DR/-DR ratio	-DR	+DR	+DR/-DR ratio	+DR/-DR ratio
2015	1.12±0.055	1.213±0.026	1.09±0.05	1.427±0.058	2.063±0.052	1.45±0.05	**
2016	2.473±0.09	2.633±0.059	1.07±0.03	2.497±0.229	3.62±0.035	1.47±0.11	*

Table S5. Statistical results of the assembled contigs, predicted genes and non-redundant genes based on one pooled metagenomic sample.

	Number	Average length (bp)	Total length (bp)	GC%
Contigs	12,914,552	903	11,667,156,729	63.80
Predicted genes	20,591,824	505	10,398,390,000	64.04
Non-redundant genes	19,803,415	513	10,159,130,000	64.02

Table S6. Statistical summary of the sequence number, assembly rate and the number of genes and reads aligned to the non-redundant gene catalogue for each of the 18 samples. The non-redundant gene catalogue was assembled by pooling all the sequence of the 18 samples.

Sample ID	Read length (bp)	No. of clean reads	Clean bases (bp)	Assembly rate (%)	Non-redundant genes No. of mapped genes	Non-redundant genes No. of mapped reads
A1A	100	117,480,406	11,748,040,600	56.04	10,310,338	54,965,354
A2A	100	118,470,002	11,847,000,200	56.67	10,337,028	55,433,789
A3A	100	120,550,898	12,055,089,800	56.45	10,398,242	56,659,382
B1A	100	119,455,864	11,945,586,400	47.31	11,285,906	43,148,752
B2A	100	107,046,614	10,704,661,400	45.88	10,652,336	37,757,100
B3A	100	103,753,796	10,375,379,600	47.17	10,481,451	36,196,590
C1A	100	114,524,026	11,452,402,600	55.80	10,146,888	50,241,613
C2A	100	119,337,936	11,933,793,600	56.18	10,323,948	52,852,549
C3A	100	118,810,426	11,881,042,600	56.56	10,321,289	53,543,050
D1A	100	117,942,668	11,794,266,800	52.87	10,157,016	50,635,220
D2A	100	119,513,002	11,951,300,200	55.16	10,161,521	50,863,018
D3A	100	117,830,492	11,783,049,200	55.89	10,109,970	50,651,374
E1A	100	110,182,168	11,018,216,800	47.29	10,422,010	37,955,635
E2A	100	108,345,846	10,834,584,600	46.68	10,397,516	37,477,146
E3A	100	107,387,492	10,738,749,200	44.79	10,377,193	37,374,868
F1A	100	112,562,758	11,256,275,800	42.87	10,319,734	36,402,068
F2A	100	115,478,306	11,547,830,600	41.15	10,457,412	37,609,372
F3A	100	106,995,852	10,699,585,200	44.16	10,175,225	34,962,464
Pooling 18 samples		2,055,668,552	205,566,855,200	50.67	19,803,415	814,729,344

Table S7. The number of genes and reads aligned to the non-redundant gene catalogue and the annotated databases for each of the 18 samples. The non-redundant gene catalogue was assembled by pooling all the sequence of the 18 samples. The taxonomic and functional databases included NCBI microbial NR database, KEGG database, eggNOG database, CAZy database and TCDB database.

	NCBI microbial NR database		KEGG database		eggNOG: all 41 databases		eggNOG: NOG+COG database		CAZy database		TCDB database	
Sample ID	No. of mapped reads	No. of mapped genes	No. of mapped reads	No. of mapped genes	No. of mapped reads	No. of mapped genes	No. of mapped reads	No. of mapped genes	No. of mapped reads	No. of mapped genes	No. of mapped reads	No. of mapped genes
A1A	49,879,058	9,195,463	36,249,602	6,336,845	152,373,278	27,322,179	45,405,786	8,232,106	4,877,840	1,177,039	1,061,593	99,273
A2A	50,222,037	9,219,764	36,533,218	6,346,793	154,002,614	27,389,215	45,788,189	8,248,889	4,906,033	1,179,821	1,065,653	99,169
A3A	51,381,713	9,268,095	37,395,195	6,380,773	157,585,081	27,544,343	46,852,802	8,296,003	5,021,335	1,187,459	1,090,556	98,062
B1A	39,712,891	10,041,281	27,675,409	6,931,778	118,931,917	30,272,773	35,115,305	9,039,693	4,626,052	1,346,438	1,135,189	67,796
B2A	34,617,024	9,539,637	23,899,226	6,550,429	103,563,870	28,693,162	30,467,843	8,547,326	4,059,683	1,277,054	984,455	76,665
B3A	33,164,228	9,418,587	22,858,613	6,453,976	98,617,719	28,219,831	29,116,865	8,416,325	3,880,717	1,253,804	942,229	79,266
C1A	45,369,035	9,182,095	31,680,066	6,120,335	136,510,444	26,811,438	40,407,341	8,036,791	5,313,758	1,197,511	1,351,167	91,058
C2A	47,931,747	9,327,930	33,875,148	6,247,605	144,622,248	27,270,527	43,003,622	8,189,610	5,606,985	1,214,121	1,438,947	88,398
C3A	48,563,787	9,318,071	34,357,100	6,243,762	147,256,641	27,285,822	43,658,855	8,187,079	5,696,948	1,215,846	1,441,582	89,036
D1A	46,309,794	9,346,521	32,823,296	6,098,602	140,441,263	26,751,316	41,436,971	8,028,062	5,325,223	1,190,419	749,823	127,880
D2A	46,485,192	9,359,741	33,041,932	6,099,512	141,450,806	26,771,064	41,683,546	8,031,994	5,334,606	1,190,501	759,230	127,546
D3A	46,239,114	9,316,054	32,791,025	6,058,747	140,788,838	26,632,908	41,454,573	7,986,228	5,315,768	1,185,497	746,204	129,446
E1A	34,838,675	9,299,353	23,698,318	6,389,884	103,074,039	27,985,184	30,401,013	8,343,446	4,167,400	1,254,986	967,274	83,016
E2A	34,432,736	9,274,427	23,408,367	6,381,064	101,742,007	27,935,215	30,026,659	8,329,767	4,122,177	1,252,327	960,179	82,909
E3A	34,382,006	9,271,374	23,528,986	6,392,768	102,023,605	27,934,792	30,078,383	8,329,537	4,095,588	1,249,478	970,097	82,463
F1A	33,264,948	9,233,410	22,778,950	6,318,509	99,752,774	27,777,638	29,268,881	8,264,593	4,023,082	1,239,609	888,425	85,827
F2A	34,435,030	9,344,806	23,584,573	6,404,239	103,139,538	28,137,128	30,282,388	8,375,103	4,162,974	1,256,491	921,440	82,869
F3A	31,992,424	9,138,702	21,959,654	6,249,037	95,730,302	27,407,285	28,149,432	8,163,004	3,859,526	1,222,087	857,297	87,176
Pooling 18 samples	743,221,439	15,031,679	522,138,678	10,304,066	2,241,606,984	46,481,097	662,598,454	13,864,917	84,395,695	1,974,785	18,331,340	309,600

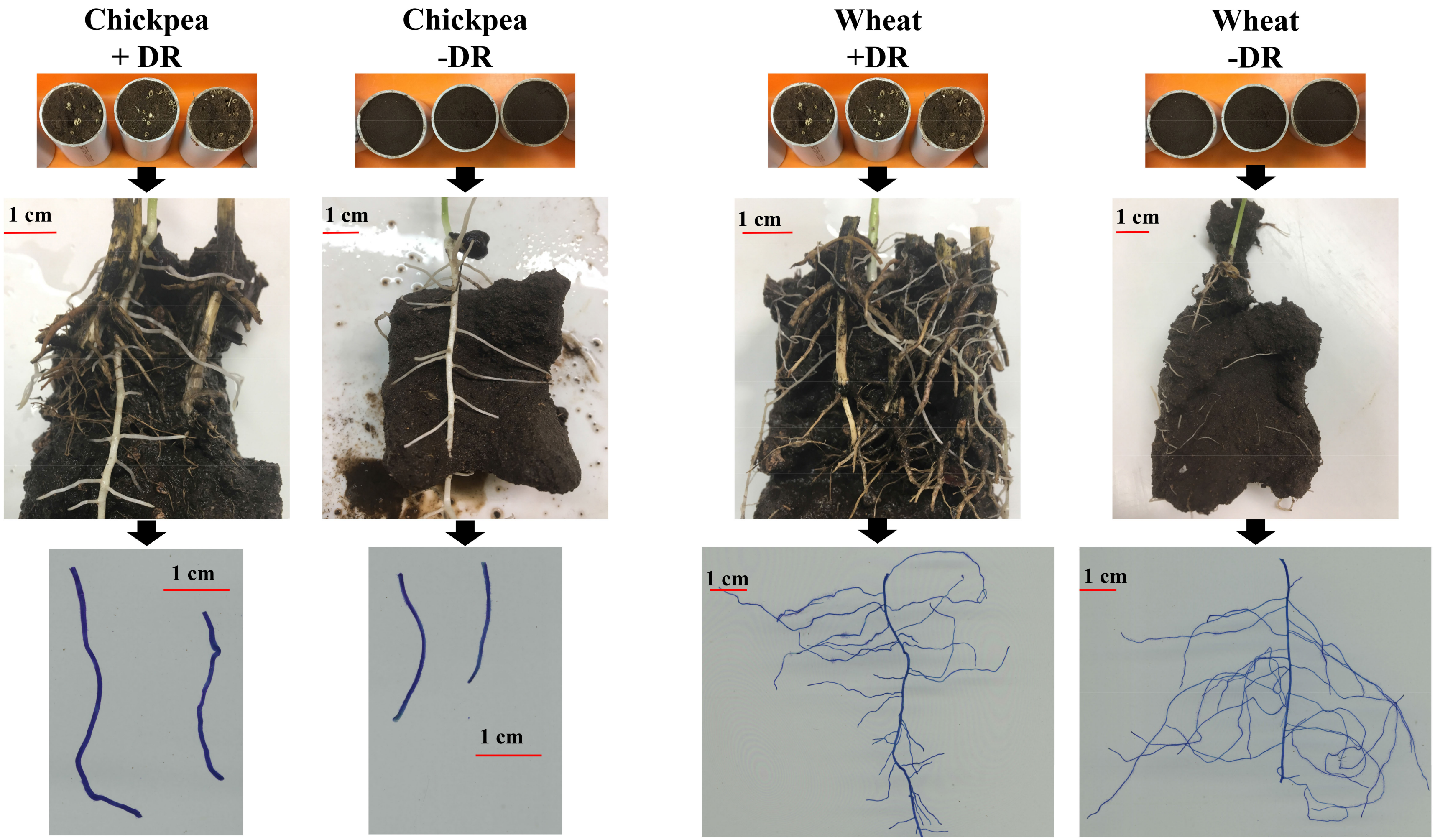

Fig S1. Pictures of fresh root-residue root contact and root distortion of wheat and chickpea under + and – decaying root (DR).

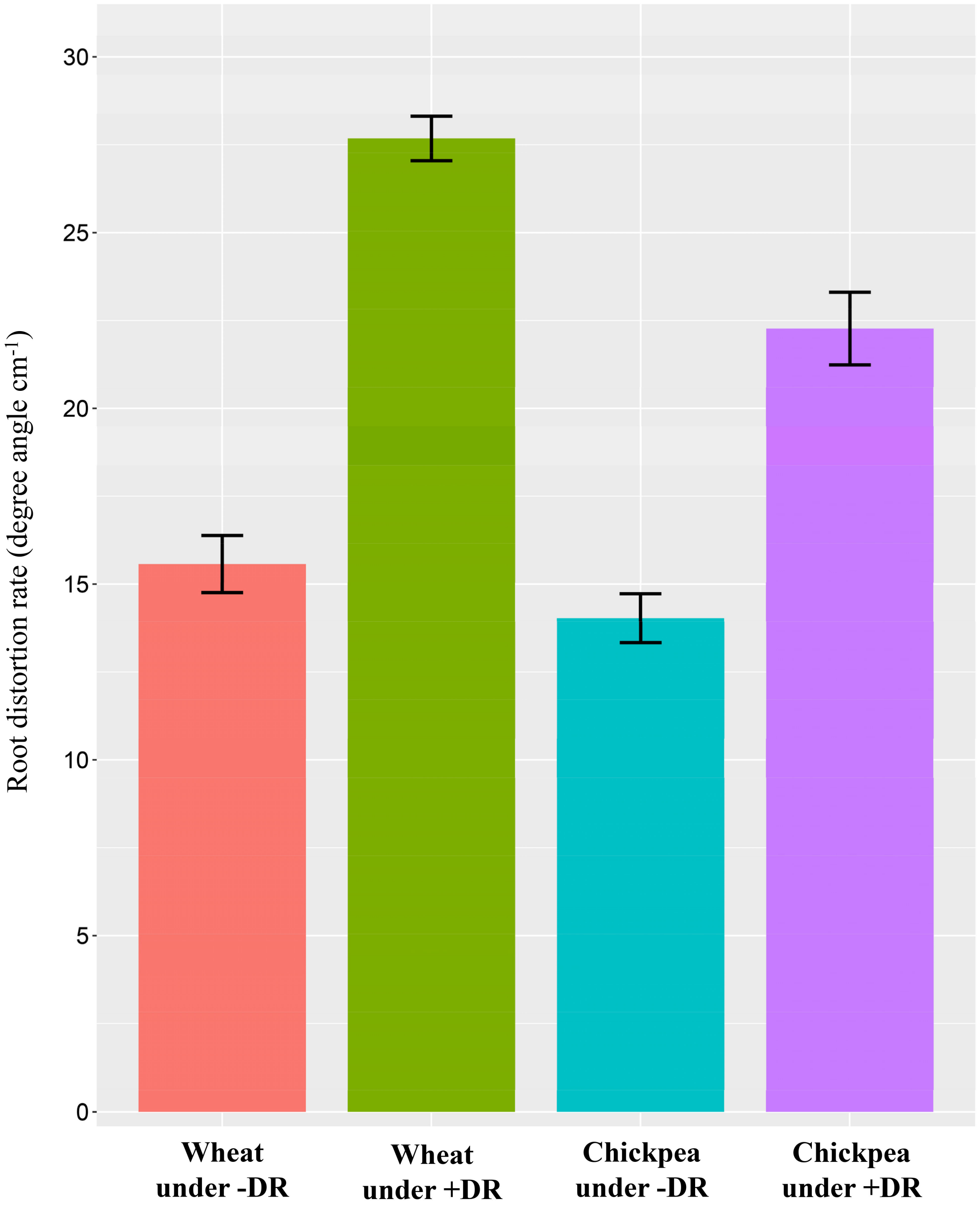

Fig S2. Root distortion rate of wheat and chickpea influenced by decaying root (DR). ANOVA test at $P < 0.05$ showed that treatment effect was significant. Bars indicate standard error at $P=0.05$.

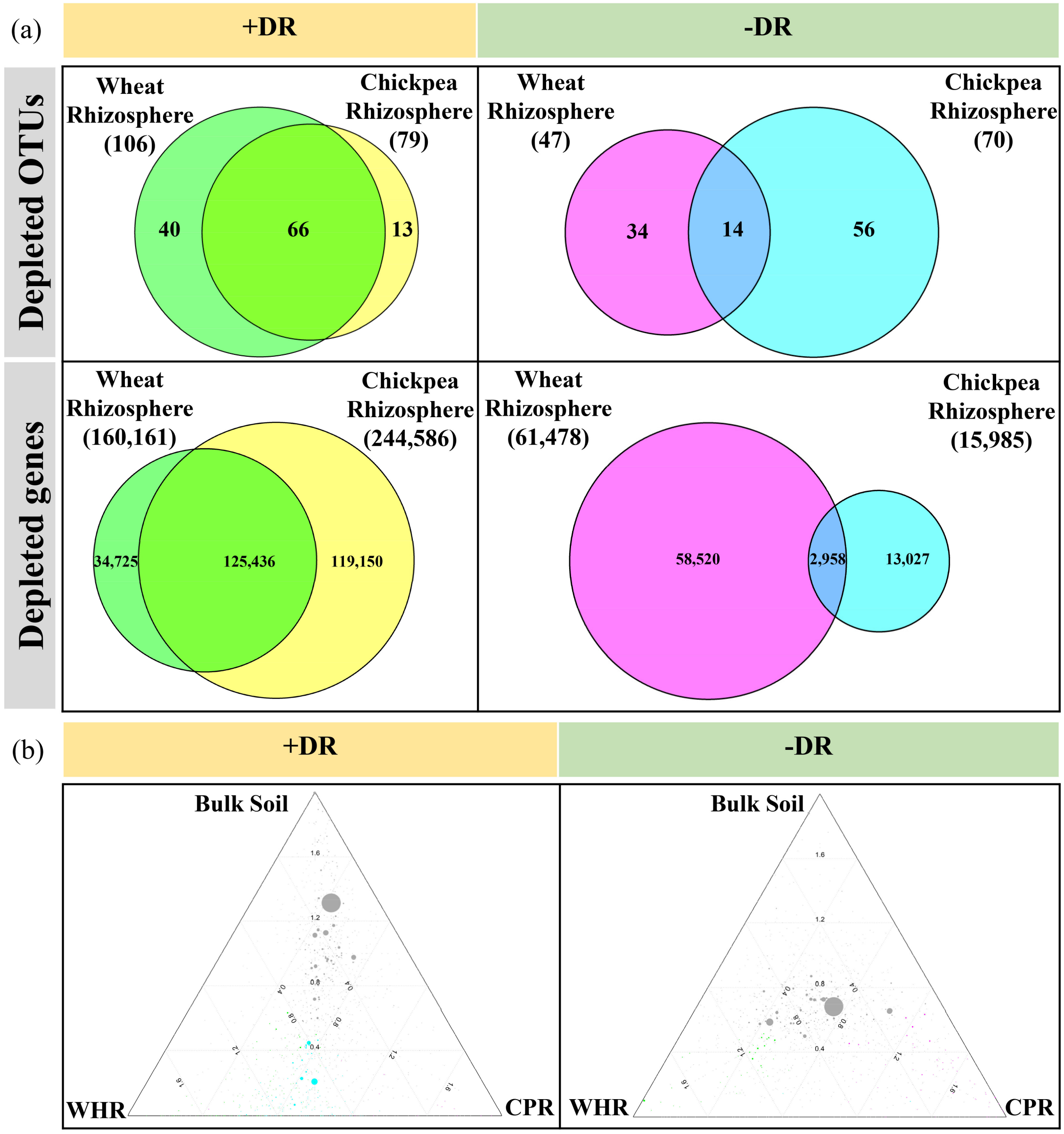

Fig S3. Comparing the functional and taxonomic structure of rhizosphere microbiome between wheat and chickpea growing under + and – decaying root (DR).

(a) the number of depleted OTUs and genes shared between wheat and chickpea under + and – DR. (b) ternary plot included all the detected OTUs between bulk soil and the rhizosphere of wheat (WHR) and chickpea (CPR) under + and – DR. Each circle indicates one OTU. The size of each circle indicates its relative abundance weighted by the average. Each circle's position is determined by the contribution of bulk soil and two plants' rhizospheres. Green circles indicate the enriched OTUs by wheat rhizosphere compared with bulk soil (log2-fold-change > 1 and the FDR adjusted p-value < 0.01). Magenta circles indicate the enriched OTUs by chickpea rhizosphere. Cyan circles indicate the enriched OTUs by both wheat and chickpea rhizosphere.

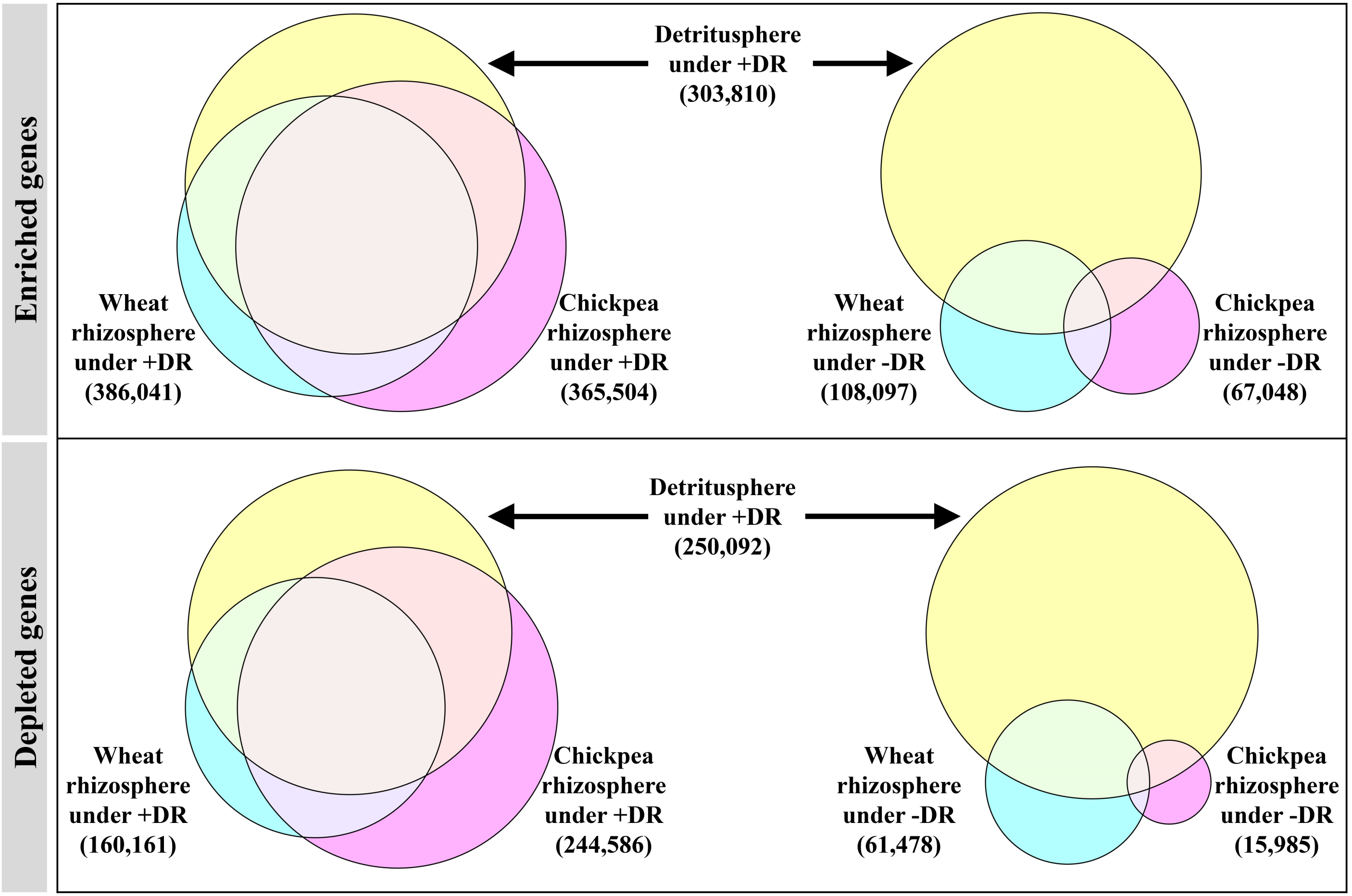

Fig S4. Comparison of differentially abundant genes between rhizosphere and detritusphere microbiome under + and – decaying root (DR). Detritusphere referred to the soils surrounding the decaying root from the unplanted control under +DR. The size of the circle area and overlapped area is proportional to the number of enriched / depleted genes.

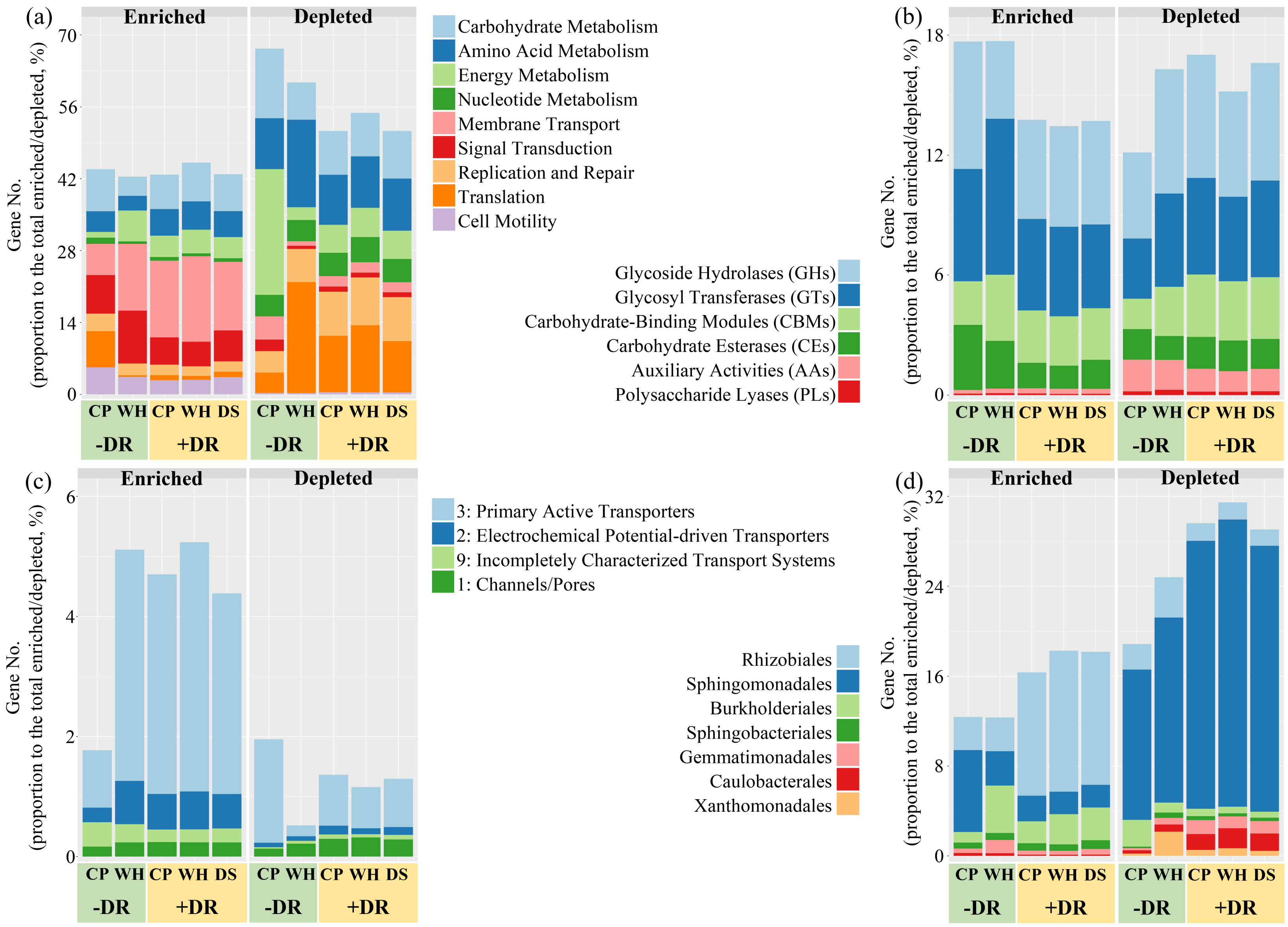
(a)
Enriched
Depleted
Gene No.
(proportion to the total enriched/depleted, %)
0
14
28
42
56
70
CP WH CP WH DS
-DR
+DR
CP WH CP WH DS
-DR
+DR
Carbohydrate Metabolism
Amino Acid Metabolism
Energy Metabolism
Nucleotide Metabolism
Membrane Transport
Signal Transduction
Replication and Repair
Translation
Cell Motility
(b)
Enriched
Depleted
Gene No.
(proportion to the total enriched/depleted, %)
0
6
12
18
Glycoside Hydrolases (GHs)
Glycosyl Transferases (GTs)
Carbohydrate-Binding Modules (CBMs)
Carbohydrate Esterases (CEs)
Auxiliary Activities (AAs)
Polysaccharide Lyases (PLs)
(c)
Enriched
Depleted
Gene No.
(proportion to the total enriched/depleted, %)
0
2
4
6
3: Primary Active Transporters
2: Electrochemical Potential-driven Transporters
9: Incompletely Characterized Transport Systems
1: Channels/Pores
(d)
Enriched
Depleted
Gene No.
(proportion to the total enriched/depleted, %)
0
8
16
24
32
Rhizobiales
Sphingomonadales
Burkholderiales
Sphingobacteriales
Gemmatimonadales
Caulobacterales
Xanthomonadales

Fig S5. Functional and taxonomic annotation of the differentially abundant genes in the rhizosphere of wheat and chickpea under + and – decaying root (DR).

The enrichment or depletion was defined as genes with higher or lower abundance in plant rhizosphere than bulk soil (log2-fold-change > 1 and the FDR adjusted p-value < 0.01), respectively. Detritusphere referred to the soils surrounding the decaying root from the unplanted control under +DR. Differentially abundant genes were annotated against (a) KEGG, (b) CAZy, (c) TADB and (d) NCBI microbial NR databases. Only groups with top average relative abundance are presented here.

(a)

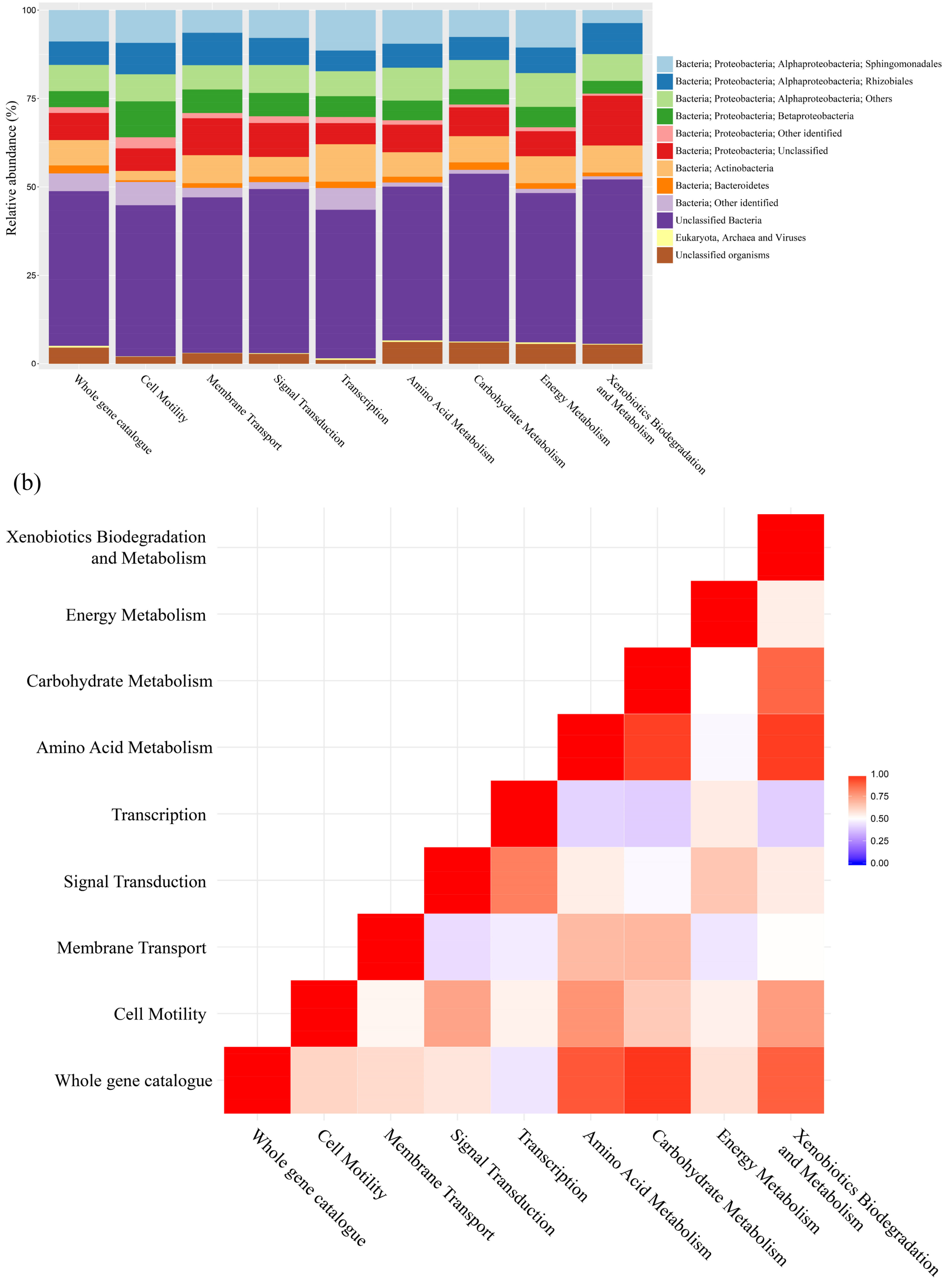

Fig S6. Taxonomic structure of rhizosphere microbiome at different metabolic pathways. (a) Taxonomic composition of the genes involved in each of eight KEGG metabolic pathways; (b) the similarities of taxonomic profile between all the samples at eight KEGG metabolic pathways. The taxonomic unit was order from lowest common ancestor analysis. Bray–Curtis distance between samples was used in the Mantel test. Correlations coefficients are presented. All the correlations were significant at $P < 0.01$.

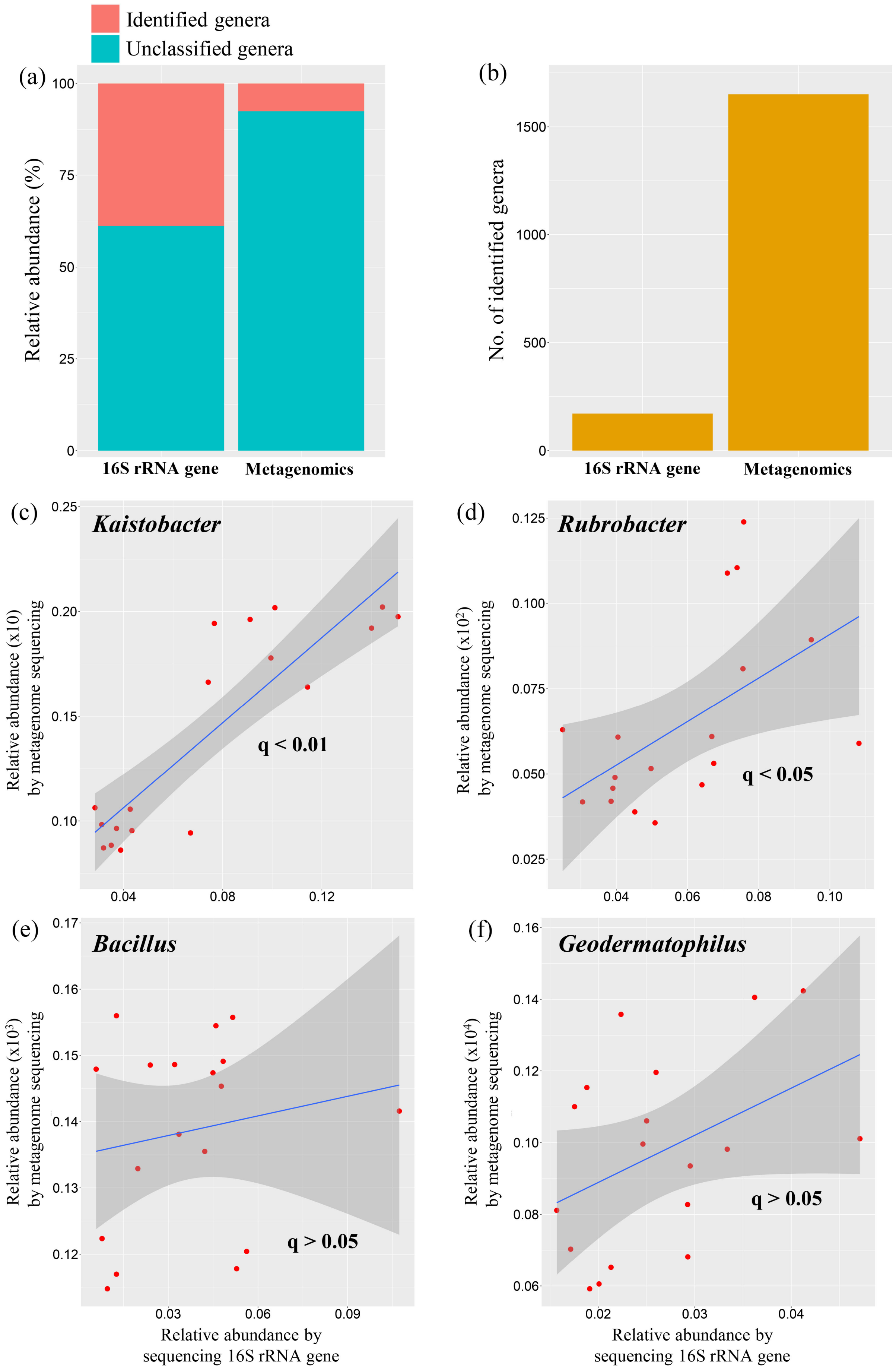
Identified genera
Unclassified genera
(a)
Relative abundance (%)
16S rRNA gene
Metagenomics
(b)
No. of identified genera
16S rRNA gene
Metagenomics
(c)
Kaistobacter
q < 0.01
Relative abundance (x10) by metagenome sequencing
(d)
Rubrobacter
q < 0.05
Relative abundance (x10²) by metagenome sequencing
(e)
Bacillus
q > 0.05
Relative abundance (x10³) by metagenome sequencing
(f)
Geodermatophilus
q > 0.05
Relative abundance (x10⁴) by metagenome sequencing
Relative abundance by sequencing 16S rRNA gene
Relative abundance by sequencing 16S rRNA gene

Fig S7. Comparison of two approaches, amplicon sequencing on 16s rRNA genes and metagenomics sequencing, to analyse microbiome taxonomic composition at genus level.
(a) proportion of sequences mapped to the identified and unclassified genera, (b) the number of identified genera, and (c-f) correlation between the two approaches for relative abundance of the dominant genera. q value (corrected P value by Bonferroni false discovery rate) was used to indicate the significant correlation.

(a)

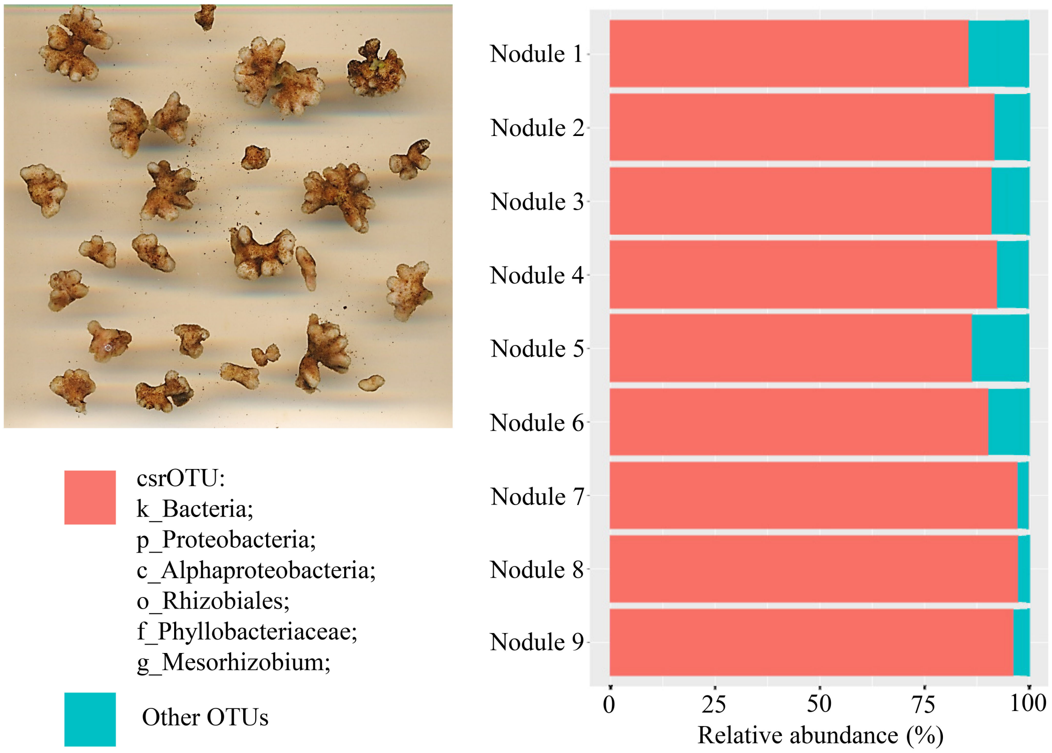

(b)

```
Query OTU:  1     TGGGGAATATTGGACAATGGGCGCAAGCCTGATCCAGCCATGCCGCGTGAGTGATGAAGG  60
                  ||||||||||||||||||||||||||||||||||||||||||||||||||||||||||||
   csrOTU:  1     TGGGGAATATTGGACAATGGGCGCAAGCCTGATCCAGCCATGCCGCGTGAGTGATGAAGG  60

Query OTU:  61    CCCTAGGGTTGTAAAGCTCTTTCAACGGTGAAGATAATGACGGTAACCGTAGAAGAAGCC  120
                  ||||||||||||||||||||||||||||||||||||||||||||||||||||||||||||
   csrOTU:  61    CCCTAGGGTTGTAAAGCTCTTTCAACGGTGAAGATAATGACGGTAACCGTAGAAGAAGCC  120

Query OTU:  121   CCGGCTAACTTCGTGCCAGCAGCCGCGGTAATACGAAGGGGGCTAGCGTTGTTCGGAATT  180
                  ||||||||||||||||||||||||||||||||||||||||||||||||||||||||||||
   csrOTU:  121   CCGGCTAACTTCGTGCCAGCAGCCGCGGTAATACGAAGGGGGCTAGCGTTGTTCGGAATT  180

Query OTU:  181   ACTGGGCGTAAAGCGCACGTAGGCGGATTGTTAAGTTAGGGGTGAAATCCCGGGCTCAA  240
                  ||||||||||||||||||||||||||||  |||||| ||||||||||||||||||||||
   csrOTU:  181   ACTGGGCGTAAAGCGCACGTAGGCGGATATTTAAGTCAGGGGTGAAATCCCGGGCTCAA  240

Query OTU:  241   CCCCGGAACTGCCTTTAATACTGGCAATCTCGAGTCCGAGAGAGGTGAGTGGAATTCCGA  300
                  |||||||||||||||| |||||||  ||||||||||||||||||||||||||||||||||
   csrOTU:  241   CCCCGGAACTGCCTTTGATACTGGGTATCTCGAGTCCGAGAGAGGTGAGTGGAATTCCGA  300

Query OTU:  301   GTGTAGAGGTGAAATTCGTAGATATTCGGAGGAACACCAGTGGCGAAGGCGGCTCACTGG  360
                  ||||||||||||||||||||||||||||||||||||||||||||||||||||||||||||
   csrOTU:  301   GTGTAGAGGTGAAATTCGTAGATATTCGGAGGAACACCAGTGGCGAAGGCGGCTCACTGG  360

Query OTU:  361   CTCGGTACTGACGCTGAGGTGCGAAAGCGTGGGGAGCAAACAGG  404
                  ||||||||||||||||||||||||||||||||||||||||||||
   csrOTU:  361   CTCGGTACTGACGCTGAGGTGCGAAAGCGTGGGGAGCAAACAGG  404
```

Fig S8. Identification of chickpea symbiotic rhizobia
(a) sequencing the clean nodules to identify the chickpea symbiotic rhizobia OTU (csrOTU).
(b) one OTU from rhizosphere matched the csrOTU best.

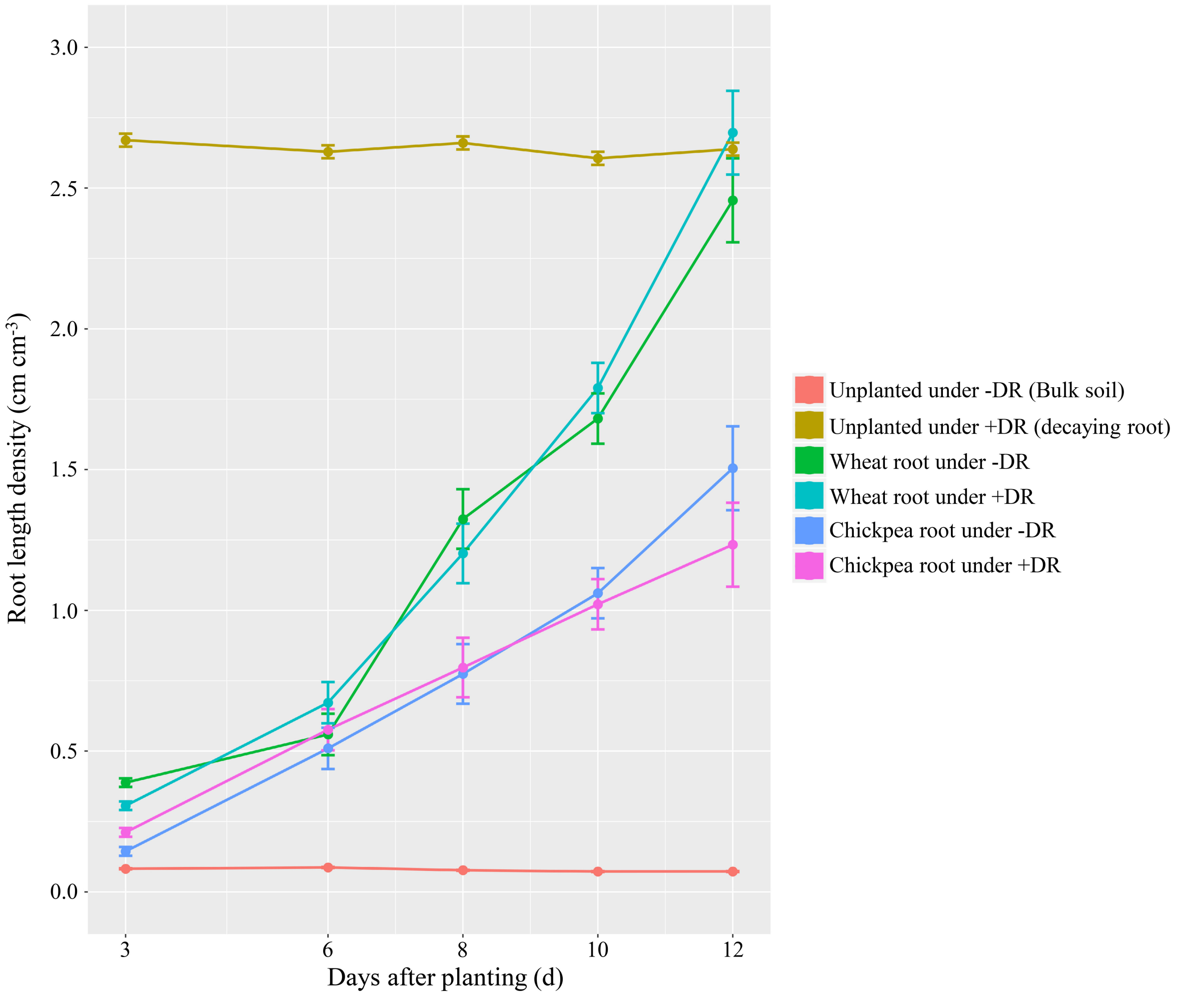

Fig S9. Root length density of wheat and chickpea under + and – decaying root (DR) changed with days after planting.

ANOVA test at $P < 0.05$ showed that treatment effect was significant. Bars indicate standard error at $P=0.05$.

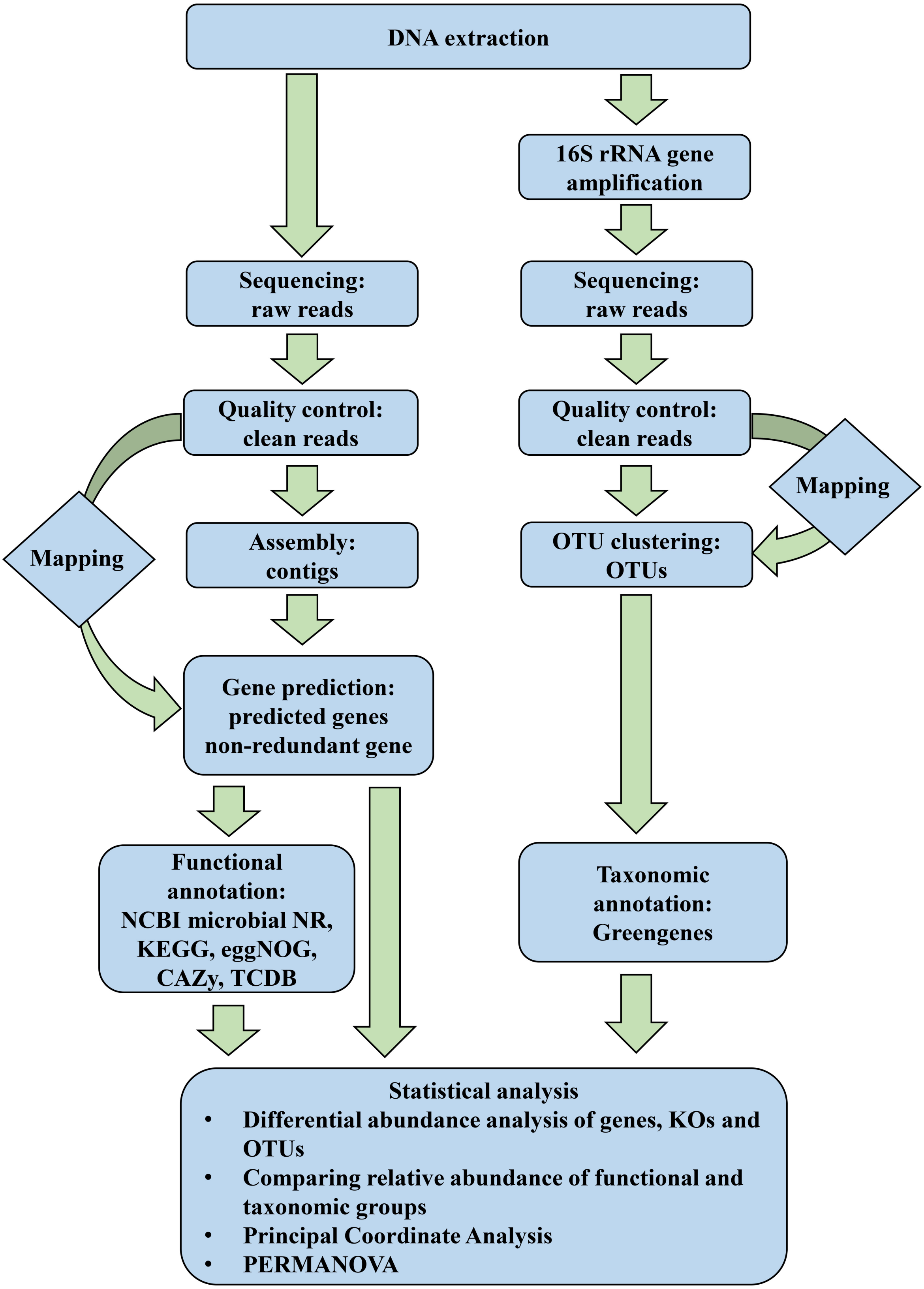

Fig S10. Flowchart of bioinformatics analysis for metagenomics sequencing and 16S rRNA gene sequencing
