# Supplementary material for: The preceding root system drives the composition and function of the rhizosphere microbiome
Source: Genome Biol. 2020 Apr 6;21:89. doi: 10.1186/s13059-020-01999-0 (PMC7137527; doi:10.1186/s13059-020-01999-0)
Supplement: Supplementary file 3 — Additional file 3: Review history. [file 13059_2020_1999_MOESM3_ESM.docx]

Review History

**First round of review**

**Reviewer 1**

**Are you able to assess all statistics in the manuscript, including the appropriateness of statistical tests used?** There are no statistics in the manuscript.

**Comments to author:**

I offer several major comments of the work.

1.      Opaque study/experimental design within and across included studies.  The results and methods sections of the work do not make clear what the experimental design was for each sub-study included in this work.  Reading the results and the methods sections, I understand that there were several treatments/conditions considered.  There was some field soil collection, a time series of seedling harvest post-sowing up to 12 days (not sure if this was in the field or in the lab), two crops (field and/or lab?), an unplanted control and two planted treatments with and without root debris, and some kind of sterile v. unsterilized treatment.  There was also a combination of field and laboratory work.  I cannot understand how many replicates or the sampling schema, and it is also unclear which soil samples (from the field or the lab) were sequenced for metagenome or amplicon sequence analysis.  Somehow there are 18 samples (from which assay/field collection?) that are presented in the first paragraph of the results.  I cannot deduce the replication for the studies.  
2.     There do not seem to be any statistical tests to support the major conclusions of the work.  The figures provide visualizations but not underlying statistical analyses (and sample sizes/replication are not reported), which would be required to make statements about differences in treatments/time/crop/tillage.
3.      Sequence analysis.  There are a lot of distinct aspects of sequence analysis for metagenomes and amplicons in this work.  The bioinformatic workflows for these analyses are not available, and the precise steps in the workflows are incomplete, and so I was unable to evaluate them.  For example, the amplicon sequence analysis does not provide many needed details (e.g., quality control/processing steps (e.g., dereplication, error correction), taxa clustering method, sequence identity of OTUs, classification/taxonomic assignment, data standardization (e.g., rarefaction).
4.     Misalignment of title/abstract with the work.  I was surprised that the study actually considers tillage v. no tillage and not is not specifically focused on the decaying rhizosphere.  I was also confused about what was considered the decaying rhizosphere - in no-tillage systems I interpreted this to be old roots (not still attached) that provide carbon -this is what the introduction suggests.  However, later there are some statements about the decaying roots of seedlings harvested a few days after sowing - and it seemed that this compartment was the focus of that particular study?  (Also, why do such young seedlings have substantially decaying roots?).  Though it was generally unclear as to the goal of the study and how the different experiments link together towards that goal, but the focus was not on decaying rhizosphere but rather till and no-till comparison, where the decaying rhizosphere is assumed to be a contributor to no-till soils.
5.     The definition of the "detritusphere" as no-till soils that have had large root pieces removed for the incubation experiment assumes that the physical pieces of the root are the only important components for decaying roots, and not the released carbon/other chemical changes that would result from no-till systems.  I am not convinced that removing root pieces and homogenizing the remaining soil would result in in a condition that is representative of a field-tilled condition.  Why not just use soil that had been tilled and not-tilled in the field for this experiment?

Other comments
The methods section is not presented in an order that makes sense for the order of the results section.  I cannot deduce which studies/experiments/field collections were relevant to which assays, and I cannot determine the experimental and study designs.

I was also confused about which samples were "pooled" (L649) and why the correlation of assembly rate (?) would be useful in assessing the pooled versus individual assemblies.  If samples are pooled, how can any statistical tests be performed to assess the impact of tillage?

I am not sure what Figure 1A shows because there was not only one sample sequenced and assembled?  Likewise, Figure 1B shows core metabolisms and I'm not sure why there would be differences in these categories over till and no-till conditions?  Figure 1C is not needed.

Figure 2A - I think that amending the ordination to include a diagonal division line is not appropriate - a statistical test like a PERMANOVA for sterilized v. non-sterilized conditions should be sufficient to determine if there is a difference.  In addition, I don't understand what was sterilize here.  The authors could use a different symbol (open/close) to indicate sterilization status.  The number of points on this figure do not align with any of the designs that I read in the methods.  Fig 2B and C will need statistical tests to determine if there are differences acr0ss root exudate profiles, and to report the same size and error bars for each treatment.  I cannot understand the experimental design.

Figure 3 - The venn diagrams make it difficult to understand the variation in the data across replicates, and it is confusing that there are different elements/treatments across panels. .  Why are unplanted data in Figure 3D and not elsewhere?  Why are bulk soils in Figure 3B and not elsewhere?  What is the detritusphere for Figure 3A and not elsewhere?

The authors lead with the importance of gene atlases, but the focus on core metabolism/conserved functions across till and no-till may be limited for allowing for understanding differences across these management strategies.

**Reviewer 2**

**Are you able to assess all statistics in the manuscript, including the appropriateness of statistical tests used?** Yes, and I have assessed the statistics in my report.

**Comments to author:**

Zhou and co-workers performed a comparative metagenomic survey to characterise the composition and functional potential of the microbial communities thriving in association with either a cereal or a legume crop grown under two simulated growing systems. They generated over 2 billion high-quality sequencing reads and they coupled this effort with an amplicon sequencing (16S rRNA gene) analysis, an exudate-degradation survey and a field trial. Collectively, their results provide relatively novel insights into the structure, function and recruitment cues of the rhizosphere microbiota and its impact on plant performance.

Although rather specific, the manuscript has the potential to impact on both basic science (molecular basis of host-microbe interactions in soil) and sustainable crop production. However, I have several concerns affecting both the concepts and the methods of this current version. Specifically:

1) The title is misleading: this is not a microbial gene catalogue but essentially a "bacterial study" since no other organisms are mentioned/discussed in the text (see also comments 3 and 4 below).
2) Lines 108-109 no characterisation of the nodulation properties and or N-fixation capabilities were performed in the study: this is the major limitation of the work and casts doubts on whether first principles can be inferred from the data. Do historical data suggest that chickpea naturally nodulate in the selected soil (i.e., the compatible N-fixer symbiont is part of the endogenous microbiota)? If yes, are the two treatments impacting on the number of nodules formed? If no are farmers amending field with the cognate inoculum (if the latter said inoculum should have been added to the treatments)? In  my vision, these are the questions to be addressed when working with legume plants and microbes. In absence of these pieces of information one could conclude that the gathered results are simply not ecologically relevant since nodulation can also affect rhizosphere communities.
3) The concept expressed in lines 140-142 is factually wrong in the light of the fact that only a fraction (39.6%) of the total reads were mapped. More prudentially, I'd state that the sequencing effort allowed the authors have an adequate coverage of the databases they interrogated, rather than the microbial metagenome of the soil.
4) The former observation trigger the question is this 60+% of the assembled reads representing other cellular organisms present in the soil profiles (e.g., fungi, oomycetes, nematodes...)? Or are they other yet unclassified bacteria? It would be interesting to generate a bacterial/fungal ratio of the DNA preparations (e.g., using 16S rRNA gene vs 18S rRNA gene qPCRs) to ascertain whether indeed Bacteria dominate the observed soil metagenomes and or the simulated tillage has an impact beyond bacterial members of the microbiome.
5) Irrespective of that it will be important discuss and/or proposing hypothesis on why, in particular under simulated no-till conditions, fungal colonization appears undetectable.
6) The description of amplicon sequencing analysis is below the minimum standard for publication: what kind of parameters were selected to retain/discard reads (e.g., minimum PHRED score), what kind of algorithm was used to map OTUs to the selected database...? For instance, the authors mentioned QIIME 1.8 which is quite outdated: the authors should consider better OTU-picking algorithms or data processing approaches developed in the past few years (e.g., DOI: 10.1128/mSystems.00003-15; doi:10.1038/ismej.2017.119). Likewise, it is not clear what kind of version has been used of the greengenes database. Finally, how many reads/sample were generated? Without these pieces of information the amplicon survey cannot be properly interpreted.
7) It is imperative for both amplicon and shotgun analyses have a copy of the script(s) used for pre-processing and generating figures/statistical analyses in an appropriate repository (e.g., https://github.com/) linked to Availability of data and material.
8) Figure 3 and Figure S6. The authors argued that on the basis of differential enrichment/depletion of OTUs and genes the rhizosphere of the crops are more or less similar. While I concur that the provided piece of evidence is necessary, per se it is not sufficient to sustain said claim: it is important to compare between rhizospheres (e.g., sNT Wheat and sNT Chickpea) to identify the OTUs and genes which are significantly different among the tested plants. In my vision, without this analysis the authors cannot accurately build the model depicted in Figure 5.
9) With 18 samples it would be more accurate depicting each one of them as an individual column in Figure 4.
10) Figure S7 suggests that the two sequencing approaches are differentially biased, therefore it is not accurate presenting them in the same framework (e.g., Fig 3). However, what is missing is a proper correlation analysis conducted by retrieving rRNA sequences from the metagenomic dataset and then processing these data in a similar manner than amplicon products. Only then, in my vision, the comparison will be meaningful.
11) I have difficulties in interpreting Table S8: if this is a field trial, I'd rather present the yield data per unit of surface (i.e., end-point data). This would allow the authors to control for the different developmental patters of each crop in the two systems and the different plant density these systems may sustain.
12) Elements of the discussion are speculative at this stage. For example
       342-348: Rhizobiales and Sphingomonadales include also pathogenic species and the N2-fixation pathway has not been reconstructed in the presented manuscript. The enrichment of these two members per se is not a proxy for the enrichment of plant growth promoting rhizobacteria.
       381-383: Not sure what is the selling point of this statement but if it has to do with the presumed reduced complexity of soil bacterial communities it is important to keep in mind that taxonomic surveys can scratch just the surface of the genetic potential of the plant-associated microbiota (e.g., 10.1016/j.chom.2018.06.011; 10.1016/j.chom.2018.06.006).
       484-489: certainly at phylum and order levels, what about at lower taxonomic ranks? This relates to comment 10 above.
End

**Authors Response**

**Point-by-point responses to the reviewers’ comments:**

Q1. Opaque study/experimental design within and across included studies. The results and methods sections of the work do not make clear what the experimental design was for each sub-study included in this work. Reading the results and the methods sections, I understand that there were several treatments/conditions considered. There was some field soil collection, a time series of seedling harvest post-sowing up to 12 days (not sure if this was in the field or in the lab), two crops (field and/or lab?), an unplanted control and two planted treatments with and without root debris, and some kind of sterile v. unsterilized treatment. There was also a combination of field and laboratory work. I cannot understand how many replicates or the sampling schema, and it is also unclear which soil samples (from the field or the lab) were sequenced for metagenome or amplicon sequence analysis. Somehow there are 18 samples (from which assay/field collection?) that are presented in the first paragraph of the results. I cannot deduce the replication for the studies.

*Reply: we agree with the reviewer’s comment, and have re-written the entire Methods and Results sections to clarify the methodologies used. The total structure of the revised Methods section is listed below. The detailed descriptions of each experiment including experimental factors, design, replicate number, plant type and growing conditions have been provided. For the key experiment for metagenomic analysis (e.g. Expt 2), the detail descriptions for each sample have been added (Additional file 1: Table S1) in the revised version of the manuscript.*

*1. Experimental design*

*2. Field site and Soil collection*

*3. Experimentation*

*3.1 Controlled environment experiments*

*(i) Experiment 1: Root developments*

*(ii) Experiment 2: Rhizosphere microbiome*

*(iii) Experiment 3: Root exudation*

*(iv) Experiment 4: Soil microbial respiration*

*(v) Experiment 5: Chickpea symbiotic rhizobia*

*3.2 Field experiment*

*4. Data analysis*

*4.1 Metagenome sequencing and bioinformatics*

*4.2 16S rRNA genes sequencing and bioinformatics*

*4.3 Statistics*

Q2. There do not seem to be any statistical tests to support the major conclusions of the work. The figures provide visualizations but not underlying statistical analyses (and sample sizes/replication are not reported), which would be required to make statements about differences in treatments/time/crop/tillage.

*Reply: we have re-written the entire Methods and Results sections. In the revised version, the detail experimental design, replicates, statistical tests and methods have been added.*

Q3. Sequence analysis. There are a lot of distinct aspects of sequence analysis for metagenomes and amplicons in this work. The bioinformatic workflows for these analyses are not available, and the precise steps in the workflows are incomplete, and so I was unable to evaluate them. For example, the amplicon sequence analysis does not provide many needed details (e.g., quality control/processing steps (e.g., dereplication, error correction), taxa clustering method, sequence identity of OTUs, classification/taxonomic assignment, data standardization (e.g., rarefaction).

*Reply: we agree with the reviewer’s comment, and have re-written the entire Methods and Results sections, including that the bioinformatic workflows for metagenomics and 16S rRNA gene analysis have been provided in Fig S9.*

Q4. Misalignment of title/abstract with the work. I was surprised that the study actually considers tillage v. no tillage and not is not specifically focused on the decaying rhizosphere. I was also confused about what was considered the decaying rhizosphere - in no-tillage systems I interpreted this to be old roots (not still attached) that provide carbon -this is what the introduction suggests. However, later there are some statements about the decaying roots of seedlings harvested a few days after sowing - and it seemed that this compartment was the focus of that particular study? (Also, why do such young seedlings have substantially decaying roots?). Though it was generally unclear as to the goal of the study and how the different experiments link together towards that goal, but the focus was not on decaying rhizosphere but rather till and no-till comparison, where the decaying rhizosphere is assumed to be a contributor to no-till soils.

*Reply: we agree with the reviewer’s comment, and realise that we had confused the reader using the terms: no-tillage, conventional tillage, + decaying root and no decaying root in the previous submitted version. We have now clarified and simplified the terminology.*

*We sampled intact soil core with decaying root (DR) in the field, and then grew wheat and chickpea in it in a controlled environment. Additionally, we grew wheat and chickpea in the same soil in the absence of decaying root. Unplanted pots in the presence and absence of DR were used as controls. We sampled the rhizosphere soil of living roots of wheat and chickpea under the two growing conditions, soil surrounding the decaying root (detritusphere) and bulk soil for analysis (Additional file 1: Table S1).*

*We have now revised the entire manuscript including all the tables and figures. In the revised version, we simplified the terminology and used + and – decaying root (+ and – DR) to indicate the growing conditions as a replacement for no-tillage and conventional tillage as used previously. The detailed descriptions of each of the experiments to the goal of the study have been provided.*

Q5. The definition of the "detritusphere" as no-till soils that have had large root pieces removed for the incubation experiment assumes that the physical pieces of the root are the only important components for decaying roots, and not the released carbon/other chemical changes that would result from no-till systems. I am not convinced that removing root pieces and homogenizing the remaining soil would result in in a condition that is representative of a field-tilled condition. Why not just use soil that had been tilled and not-tilled in the field for this experiment?

*Reply: Over the long-term, no-till can alter soil properties, such as organic matter, compared with tilled systems. In the short-term, the difference between tillage and notillage is the existence of the physical pieces of the decaying root as the reviewer mentioned. Here we would like to highlight that the decaying root can direct the growth of living roots in a “sense-by-growth” mechanisms, increase the living root – residue root contact, and change the rhizosphere microbiome (living root) to be more similar to the detritusphere microbiome (decaying root). If we sampled soil that had been tilled and not-tilled in the field for the experiment, we could not distinguish if the changes of the rhizosphere microbiome were due to differences in the soil physiochemical properties or due to the existence of the decaying root, so we needed to remove roots to provide a clearer treatment effect.*

Q6. The methods section is not presented in an order that makes sense for the order of the results section. I cannot deduce which studies/experiments/field collections were relevant to which assays, and I cannot determine the experimental and study designs.

*Reply: please see our reply to reviewer 1 Q1 above.*

Q7. I was also confused about which samples were "pooled" (L649) and why the correlation of assembly rate (?) would be useful in assessing the pooled versus individual assemblies. If samples are pooled, how can any statistical tests be performed to assess the impact of tillage?

*Reply: Samples were only pooled together for metagenomic de novo assembly to establish the gene catalogue. The sequences from each sample were independently mapped to the gene catalogue. The relative abundance of genes in each sample was independent, and used for statistics. We have removed the section comparing the pooled versus individual assemblies. The bioinformatic workflows for metagenomic analyses have been provided in Fig S9.*

Q8. I am not sure what Figure 1A shows because there was not only one sample sequenced and assembled? Likewise, Figure 1B shows core metabolisms and I'm not sure why there would be differences in these categories over till and no-till conditions? Figure 1C is not needed.

*Reply: we have restructured the figure to better represent the gene catalogue (see Fig 6). We showed the rarefaction curves to highlight that saturation was reached, and the sequencing depth was adequate to cover the microbial metagenome. We also showed the functional and taxonomic distribution of the gene catalogue based on gene number.*

Q9. Figure 2A - I think that amending the ordination to include a diagonal division line is not appropriate - a statistical test like a PERMANOVA for sterilized v. non-sterilized conditions should be sufficient to determine if there is a difference. In addition, I don't understand what was sterilize here. The authors could use a different symbol (open/close) to indicate sterilization status. The number of points on this figure do not align with any of the designs that I read in the methods. Fig 2B and C will need statistical tests to determine if there are differences acr0ss root exudate profiles, and to report the same size and error bars for each treatment. I cannot understand the experimental design.

*Reply: we agree with the reviewer’s comment, and have redone the figures of root exudation (Fig 1a). We have used different symbol (open/close) to indicate the sterilization status. We used sterilized and non-sterilized conditions here to estimate the degradation of root exudates by microbiome (Fig 1b). Statistical tests were conducted to compare different sample niches for major groups of root exudates. Lsd has been marked in the figure and P values have been provided in the text. We have rewritten the entire Methods and Results sections to describe the experimental design clearly. Please also see our reply to review1, Q1.*

Q10. Figure 3 - The venn diagrams make it difficult to understand the variation in the data across replicates, and it is confusing that there are different elements/treatments across panels. . Why are unplanted data in Figure 3D and not elsewhere? Why are bulk soils in Figure 3B and not elsewhere? What is the detritusphere for Figure 3A and not elsewhere?

*Reply: we have restructured the related figures, and rewritten the Methods and Results sections.*

*The Venn diagrams are based on the differential abundance analysis. We run a differential abundance analysis by using unplanted bulk soil with no decaying root as the control to compare with other niches for each OTU and non-redundant gene. The enriched and depleted OTUs and non-redundant genes for each niche were defined based on log2-fold-change > 1 and the FDR adjusted p-value < 0.01 to bulk soil using 3 replicates. And then only the enriched and depleted OTUs and non-redundant genes were used to create the Venn diagrams. Therefore, bulk soil and replicates cannot be presented in the Venn diagrams.*

*Ternary plot and Principal Coordinate Analysis (PCoA) are based on the abundance of all the OTUs, KOs and non-redundant genes across all the samples (6 niches x 3 reps). Therefore, the ternary plot showed the average values of the replicates. The PCoA plot showed the Bray-Curtis distance between all 18 samples, and the statistical test for Bray-Curtis distance is shown in Table 1 of the revised manuscript.*

Reviewer #2:

Q1. The title is misleading: this is not a microbial gene catalogue but essentially a "bacterial study" since no other organisms are mentioned/discussed in the text (see also comments 3 and 4 below).

*Reply: our soil metagenome included all the microorganisms, bacteria and fungi. We can do a fungi function and taxonomic analysis between sampled niches, but we chose not to do that, because compared with bacteria, the diversity and size of fungal communites were quite low. Please also see the reply to reviewer 2’s Q4. In addition to this, we have now redeveloped a title that better highlights the biological meaning of our results.*

Q2. Lines 108-109 no characterisation of the nodulation properties and or N-fixation capabilities were performed in the study: this is the major limitation of the work and casts doubts on whether first principles can be inferred from the data. Do historical data suggest that chickpea naturally nodulate in the selected soil (i.e., the compatible N-fixer symbiont is part of the endogenous microbiota)? If yes, are the two treatments impacting on the number of nodules formed? If no are farmers amending field with the cognate inoculum (if the latter said inoculum should have been added to the treatments)? In my vision, these are the questions to be addressed when working with legume plants and microbes. In absence of these pieces of information one could conclude that the gathered results are simply not ecologically relevant since nodulation can also affect rhizosphere communities.

*Reply: we agree with the reviewer’s comment, and added a new experiment which we were planning to publish separately. In the newly added experiment, the OTU sequence of chickpea symbiotic rhizobia (csrOTU) was identified by sequencing the cleaned nodules of chickpea after growing in the same soil for 4 weeks. Then we looked for the csrOTU among all of the OTUs in the rhizosphere niche experiment, and one OTU was highly matched. So we compared the abundance of csrOTU among all the sampled niches. Please see Fig S7, Fig 5 and related descriptions in the main manuscript of the revised version.*

Q3. The concept expressed in lines 140-142 is factually wrong in the light of the fact that only a fraction (39.6%) of the total reads were mapped. More prudentially, I'd state that the sequencing effort allowed the authors have an adequate coverage of the databases they interrogated, rather than the microbial metagenome of the soil.

*Reply: we agree with the reviewer’s comment, as gene prediction was carried out on MetaGeneMark based on NCBI Reference Sequence Database. Please see our modifications at the line 321-323 in the revised version of manuscript.*

Q4. The former observation trigger the question is this 60+% of the assembled reads representing other cellular organisms present in the soil profiles (e.g., fungi, oomycetes, nematodes...)? Or are they other yet unclassified bacteria? It would be interesting to generate a bacterial/fungal ratio of the DNA preparations (e.g., using 16S rRNA gene vs 18S rRNA gene qPCRs) to ascertain whether indeed Bacteria dominate the observed soil metagenomes and or the simulated tillage has an impact beyond bacterial members of the microbiome. 5) Irrespective of that it will be important discuss and/or proposing hypothesis on why, in particular under simulated no-till conditions, fungal colonization appears undetectable.

*Reply: We used the method of lowest common ancestor to annotate the gene catalogue for taxonomy, e.g. if one gene is detected in more than one bacteria phyla in NCBI microbial NR database, this gene is annotated as “unidentified bacteria”. So we have revised the pie chart for taxonomy. In the revised chart, “unidentified bacteria” is merged into bacteria. We did qPCR for 16S rRNA gene and ITS gene, and demonstrated the low abundance of fungi community in our samples. Please see our modifications in Table 1, line 163-167 and line 331-335 in the revised version of the manuscript*

Q5. The description of amplicon sequencing analysis is below the minimum standard for publication: what kind of parameters were selected to retain/discard reads (e.g., minimum PHRED score), what kind of algorithm was used to map OTUs to the selected database...? For instance, the authors mentioned QIIME 1.8 which is quite outdated: the authors should consider better OTU-picking algorithms or data processing approaches developed in the past few years (e.g., DOI: 10.1128/mSystems.00003-15; doi:10.1038/ismej.2017.119). Likewise, it is not clear what kind of version has been used of the greengenes database. Finally, how many reads/sample were generated? Without these pieces of information the amplicon survey cannot be properly interpreted.

*Reply: we have rewritten the bioinformatics section for amplicon sequencing analysis by providing more detailed information as required by the reviewer, and added a flow chart (Fig S9) of the analysis procedure. We understand that there are some updated microbiome sequence inference methods, such as amplicon sequence variant (ASV) method, however, the argument of relevant advantages and disadvantages between OTU and ASV methods continues (e.g. a good summary from Noah Fierer’s lab: http://fiererlab.org/2017/05/02/lumping-versus-splitting-is-it-time-for-microbialecologists-to-abandon-otus/). Here, we would like to focus more on the biological meaning of our analysis.*

Q6 It is imperative for both amplicon and shotgun analyses have a copy of the script(s) used for pre-processing and generating figures/statistical analyses in an appropriate repository (e.g., https://github.com/) linked to Availability of data and material.

*Reply: currently we are applying the accessible online storage for the reader to download the R code we used for statistics. We plan to have it ready before the manuscript is published.*

Q7 Figure 3 and Figure S6. The authors argued that on the basis of differential enrichment/depletion of OTUs and genes the rhizosphere of the crops are more or less similar. While I concur that the provided piece of evidence is necessary, per se it is not sufficient to sustain said claim: it is important to compare between rhizospheres (e.g., sNT Wheat and sNT Chickpea) to identify the OTUs and genes which are significantly different among the tested plants. In my vision, without this analysis the authors cannot accurately build the model depicted in Figure 5.

*Reply: we agree with the reviewer’s comment, and have added the relative abundance of sequences in microbial gene groups and taxonomic genus between different niches (Fig 4).*

Q8 With 18 samples it would be more accurate depicting each one of them as an individual column in Figure 4.

*Reply: due to the limited space required from the journal (all the subfigures should be in one page), we showed the average values in the heatmap, but presented the mean value and standard error (SE) in Additional file 3.*

Q9 Figure S7 suggests that the two sequencing approaches are differentially biased, therefore it is not accurate presenting them in the same framework (e.g., Fig 3). However, what is missing is a proper correlation analysis conducted by retrieving rRNA sequences from the metagenomic dataset and then processing these data in a similar manner than amplicon products. Only then, in my vision, the comparison will be meaningful.

*Reply: we have removed this section, and focused on the biological meaning of our analysis, as many different data have been collected in present study.*

Q10 I have difficulties in interpreting Table S8: if this is a field trial, I'd rather present the yield data per unit of surface (i.e., end-point data). This would allow the authors to control for the different developmental patters of each crop in the two systems and the different plant density these systems may sustain.

*Reply: we have rewritten the entire Methods and Results sections. Please see our reply to reviewer 1’s Q1. As the reviewer advised, we have provided the grain yield data in Additional file 1: Table S4.*

Q11. 342-348: Rhizobiales and Sphingomonadales include also pathogenic species and the N2-fixation pathway has not been reconstructed in the presented manuscript. The enrichment of these two members per se is not a proxy for the enrichment of plant growth promoting rhizobacteria.

*Reply: we have shown the taxonomy at the genus level (Fig 4), and discussed the roles of the bacterial genus.*

Q12 381-383: Not sure what is the selling point of this statement but if it has to do with the presumed reduced complexity of soil bacterial communities it is important to keep in mind that taxonomic surveys can scratch just the surface of the genetic potential of the plant-associated microbiota (e.g., 10.1016/j.chom.2018.06.011; 10.1016/j.chom.2018.06.006).

*Reply: Delgado-Baquerizo et al (Science. 2018, 359:320-325) demonstrated that over 40% of bacterial abundance was explained by only 2% of bacterial groups (about 500 phylotypes). This study was based on OTUs. The article 10.1016/j.chom.2018.06.011 as advised by the reviewer showed that the high diversity of different strains within one OTU. As it is difficult to define the complexity based on the number of OTUs, we have removed this section from the Discussion.*

Q13 484-489: certainly at phylum and order levels, what about at lower taxonomic ranks? This relates to comment 10 above.

*Reply: we have shown the taxonomy at the genus level (Fig 4), and discussed the roles of the bacteria genus.*

**Second round of review**

**Reviewer 2**

I found the revised version of Zhou et al., clearer than the original one (in particular Additional File 2: Figure S9 is extremely helpful). The vast majority of my comments have properly been addressed. Crucially, the authors have performed new experimentations and/or provided new data to support their conclusions. I have a few minor points.

Q1: Still not fascinated by the title I think does not convey the message in an effective way, what about: A 'root legacy' drives the composition and function of the rhizosphere microbiome. But this isn't my major point.

Q2-Q3-Q4-Q5: OK, thanks for having taken action on these points.

Q6: please consider public repository for your scripts, e.g. GitHub;

Q7-Q8:OK;

Q9: Still, I think that this comparison will be interesting just to gauge the overlap between the two methodologies

Q10-13: OK.
#End

**Authors Response**

**Point-by-point responses to the reviewers’ comments:**

**Reply:** we have conducted a brief comparison between metagenomic and amplicon sequencing. Please see the newly added Fig S7, and the related descriptions, lines 315-324 in the 2^nd^ revised manuscript with track changes.

**Reply:** as suggested by the editor, we have uploaded the source code to GitHut under an open source license, and to zenodo with DOI reference.

Please see

“The original data and source code for data analysis are available in the GitHub repository licensed under GNU GPLv3 https://github.com/DentonLab/decaying-root-microbiome [92], and deposited in Zenodo with DOI [https://doi.org/10.5281/zenodo.3692465 [93](https://doi.org/10.5281/zenodo.3692465%20%5b93)].” line 880 to 882 in the 2^nd^ revised manuscript with track changes.

and we have added references as

92.	Zhou Y, Coventry DR, Gupta VVSR, Fuentes D, Merchant A, Kaiser BN, Li J, Wei Y, Liu H, Wang Y, et al: DentonLab/decaying-root-microbiome. Github; 2020. <https://github.com/DentonLab/decaying-root-microbiome>

93.	Zhou Y, Coventry DR, Gupta VVSR, Fuentes D, Merchant A, Kaiser BN, Li J, Wei Y, Liu H, Wang Y, et al: DentonLab/decaying-root-microbiome. Zenodo; 2020. <https://doi.org/10.5281/zenodo.3692465>
